# Suppressing NK Cells by Astragaloside IV Protects Against Acute Ischemic Stroke in Mice *Via* Inhibiting STAT3

**DOI:** 10.3389/fphar.2021.802047

**Published:** 2022-02-03

**Authors:** Shichun Li, Baokai Dou, Shi Shu, Luyao Wei, Shiguo Zhu, Zunji Ke, Zhifei Wang

**Affiliations:** ^1^ School of Basic Medical Sciences, Shanghai University of Traditional Chinese Medicine, Shanghai, China; ^2^ Academy of Integrative Medicine, Shanghai University of Traditional Chinese Medicine, Shanghai, China

**Keywords:** natural killer cells, brain ischemia, astragaloside IV, oxygen-glucose deprivation, signal transducer and activator of transcription 3

## Abstract

Natural killer (NK) cells, a key member of innate lymphocytes, are a promising immunotherapeutic target for ischemic stroke. Astragaloside IV (ASIV) is isolated from *Astragalus mongholicus Bunge* (Fabaceae), a herbal medicine possessing immunomodulatory ability. This study investigated the effect of ASIV on NK cells during the acute stage of brain ischemic injury in a mouse model of middle cerebral artery occlusion (MCAO). MCAO mice treated with ASIV had better functional outcomes, smaller brain infarction and less NK cell brain infiltration. NK cell depletion echoed the protective effect of ASIV. Notably, ASIV did not enhance the protective effect of NK cell depletion against brain ischemic injury. ASIV inhibited glial cell-derived CCL2-mediated chemotaxis to prevent post-ischemic NK cell brain recruitment. Meanwhile, ASIV also abrogated NK cell-mediated cytolytic killing of neurons subjected to oxygen-glucose deprivation and suppressed NK cell-derived IFN-γ and NKG2D expression in the ischemic brain. The inhibitory effect of ASIV on NK cell brain infiltration and activation was mimicked by cryptotanshinone, a STAT3 inhibitor. There was no additive effect when ASIV and cryptotanshinone were used together. In conclusion, ASIV inhibits post-ischemic brain infiltration and activation of NK cells through STAT3 suppression, and this inhibitory effect of ASIV on NK cells plays a key role in its protection against acute ischemic brain injury. Our findings suggest that ASIV is a promising therapeutic candidate in NK cell-based immunotherapy for the treatment of acute ischemic stroke and pave the way for potential clinical trials.

## Introduction

Ischemic stroke accounts for approximately 80% of all strokes and is associated with high morbidity and mortality worldwide. Recombinant tissue plasminogen activator (rtPA) is the only FDA-approved intervention for ischemic stroke. Unfortunately, only a minority of stroke patients is eligible for rtPA treatment due to its narrow therapeutic time window and the potential risk of intracranial hemorrhage (Fonarow et al., 2011). Therefore, it is urgent to develop new treatments for ischemic stroke. Immune responses closely participate in all stages of ischemic cascade, from acute injury to long-term recovery, and shape the outcome of stroke (Iadecola and Anrather, 2011; Levard et al., 2020). Upon the occurrence of brain ischemia, breached blood-brain barrier (BBB) allows infiltration of large amount of peripheral immune cells to the brain parenchyma (Planas, 2018). Among them, natural killer (NK) cells are one of the first responders.

NK cells are key members of the innate immune system. They possess the ability of cytotoxicity without prior sensitization and rapid production of cytokines, predominantly interferon-γ (IFN-γ) (Vivier et al., 2008). Additionally, NK cells can regulate the adaptive immune response via modulating T cell functions (Crouse et al., 2015). Given the prompt response and unique immunology features of these cells, NK cell has been a promising candidate for cancer immunotherapy (Shimasaki et al., 2020). Moreover, NK cells have also received growing attentions as a promising target in the treatment of ischemic stroke (Gan et al., 2014; Zhang et al., 2014; Chen et al., 2019).

Infiltration of NK cells is observed in the postmortem brain of ischemic stroke patients and peaks at 2–5 days after stroke onset (Gan et al., 2014; Zhang et al., 2014). In mouse models of middle cerebral artery occlusion (MCAO), NK cells infiltrate to the ischemic brain as early as 3 h and peak at 12–72 h after MCAO (Gan et al., 2014; Zhang et al., 2014). Those infiltrated NK cells are adjacent to ischemic neurons in the penumbra area (Gan et al., 2014). Further studies have demonstrated that NK cells can directly kill ischemic neurons, augment neuroinflammation through release of IFN-γ, enhance ischemic neuronal excitability and synaptic excitatory transmission, and exacerbate BBB disruption (Gan et al., 2014; Zhang et al., 2014). After MCAO, *Rag2* ^*−/−*^ *rc* ^*−/−*^ mice that lack NK, T, NKT and B cells have smaller infarct size and less neurological deficits than *Rag2* ^*−/−*^ mice who lack T, NKT and B cells (Gan et al., 2014). Furthermore, NK cell depletion using anti-NK1.1 antibody ameliorates brain infarction and neurological deficits in MCAO mice (Gan et al., 2014; Zhang et al., 2014). Those findings indicate the detrimental role of NK cells, which may be independent of T, NKT and B cells, in ischemic stroke. Hence, targeting NK cells may provide promising strategies for ischemic stroke therapy (Chen et al., 2019).

A growing body of evidence shows that natural products have immunoregulatory effects and NK cell activation can be regulated by natural compounds (Grudzien and Rapak, 2018; Xu et al., 2020). Astragaloside IV (ASIV), a lanolin alcohol-shaped tetracyclic triterpenoid saponin with high polarity, is isolated from the herbal medicine *Astragalus mongholicus Bunge* (Fabaceae). *Astragalus* injection, a traditional Chinese medicine injection in which ASIV is the main component, is used to treat cardiovascular diseases, such as dilated cardiomyopathy with heart failure and combined coronary heart disease with heart failure in China (Wei et al., 2021; Cao et al., 2022). We have previously shown that ASIV treatment for 7 days can reduce brain infarction and ameliorate functional deficits in MCAO rats (Dou et al., 2021). In a long term study, there is no difference in body weight between control and high-fat diet-fed mice treated with ASIV for 13 weeks (Wu et al., 2016). Hence, the toxic effect of ASIV is limited within the safe dosage range. ASIV has multiple neuroprotective effects against brain ischemia, including anti-apoptosis, anti-oxidative stress and anti-inflammation (Wang et al., 2017). In addition, ASIV possesses immunomodulatory abilities. Our previous work has shown that ASIV can regulate differentiation and apoptosis of activated CD4^+^ T cells (Yang et al., 2019). It can also increase proliferation of T and B cells and antibody production (Wang et al., 2002). In the present study, we found that ASIV can suppress post-ischemic infiltration and activation of NK cells in the ischemic brain of MCAO mice, which may eventually contribute to the beneficial effects of ASIV against acute brain ischemic injury. These inhibitory effects of ASIV on NK cells may depend on signal transducer and activator of transcription 3 (STAT3) suppression. Our findings confirm the detrimental role of NK cells during the acute stage of brain ischemia, and suggest that ASIV could be potentially applied in NK cell–based immunomodulatory therapy for acute ischemic stroke treatment.

## Materials and Methods

### MCAO Surgery and Drug Treatment

Male C57BL/6 mice (23–26 g) were purchased from Vital River Laboratory Animal Technology Co., Ltd. (Beijing, China). Mice were subjected to right MCAO for 45 min followed by reperfusion for 24 h as previously described (Wang et al., 2012; Li et al., 2020). Regional cerebral blood flow (1 mm posterior, 4 mm lateral to the bregma) was monitored using a laser Doppler flowmetry (Powerlab ML191, ADInstruments, Australia). MCAO mice were randomized to ASIV (purity >98%, Tongtian Biotechnology, Shanghai, China) or vehicle treatment. ASIV (20 or 40 mg/kg, *i.p.*) was injected immediately after reperfusion and 12 h later. We did not have a positive control group, because there is no known drug possessing NK cell–based immunomodulatory effect against brain ischemic injury. For NK cell depletion, anti-NK1.1 monoclonal antibody (PK136, 10 μg/g, *i.p.*, Bioxcell, NH, United States) was administrated at 24 h before MCAO surgery. Cryptotanshinone (CT, Absin, China) was used to inhibit STAT3 phosphorylation. CT (50 mg/kg, p.o.) was administrated once daily for 7 days before MCAO and immediately after reperfusion on the day of surgery.

### Behavioral Tests

All behavioral tests were conducted by researchers blinded to the treatment details. The accelerating rotarod test and neurological deficit score were assessed at 24 h after reperfusion as previously described (Li et al., 2020).

### Brain Infarct Volume Measurement

Six 1-mm coronal brain sections were stained with 2% 2,3,5-triphenyltetrazolium chloride (TTC, Sigma, United States) and fixed in 10% formaldehyde. The infarction in white was analyzed using ImageJ.

### Immunohistochemistry and Immunofluorescence Staining

Free-floating sections (30 μm) were incubated with primary antibodies against NKp46 (1:100, BD Bioscience), CCL2 (1:1,000, R&D systems) or GFAP (1:400, Abcam) at 4°C overnight. For immunohistochemistry, brain sections were incubated with the biotinylated antibody, followed by the horseradish peroxidase and DAB (Yeasen, Shanghai, China). For immunofluorescence staining, brain sections were incubated with Alexa Fluor 488 or 594-conjugated secondary antibodies (1:400, Jackson ImmunoResearch). Immunolabeling signals were captured by Zeiss AXIO Imager M2 microscope and Leica SP8 Confocal Microscope.

### Quantitative Real-Time PCR

RNA extraction and real-time PCR were performed as previously described (Li et al., 2020). The primers for CCL2 were 5′-GTG​CTG​ACC​CCA​AGA​AGG​AAT​G-3’ (forward) and 5′-TGA​GGT​GGT​TGT​GGA​AAA​GGT​AGT​G-3’ (reverse). The relative mRNA expression was normalized against β-actin and presented as 2^−ΔΔCT^.

### Flow Cytometry

Single-cell suspensions were prepared as previously described (Dou et al., 2018). For detection of NK cells and their NKG2D expression, cells were stained with FITC-conjugated anti-NK1.1 (BD Bioscience), APC-conjugated anti-CD3 (BioLegend), PE-conjugated anti-NKG2D (BioLegend) and corresponding isotype controls. For detection of NK cell-produced IFN-γ, firstly, 1.0×10^6^ cells were activated *in vitro* with 2 μl Leukocyte Activation Cocktail (BD Bioscience) for 5 h at 37°C, followed by incubation with FITC-conjugated anti-NK1.1, APC-conjugated anti-CD3 for 30 min. Then, cells were permeabilized and fixed using Cytofix/Cytoperm Soln Kit (BD Bioscience) for 30 min, followed by incubation with PE-conjugated anti-IFN-γ (BD Bioscience). FACS was performed on BD FACS Calibur flow cytometer (BD Biosciences, NJ, United States) or CytoFLEX (BECKMAN COULTER, United States). Data were analyzed using FlowJo (version 10, United States). For analysis of NK cell infiltration, the lymphocyte population was gated from the general diagram of FSC-SSC. The negative control was then determined from cells stained with isotype controls. The location of CD3^+^ cells was determined by CD3-stained cells, and that of NK1.1^+^ cells was distinguished by NK1.1-stained cells. The percentage of NK1.1^+^CD3^−^ cells in the population was recorded.

### ELISA

The protein expression of IFN-γ in the ipsilateral cortex were detected using a mouse IFN-γ ELISA kit (Dakewei, China) according to the manufacturer’s instructions.

### Western Blotting

The protein levels of phospho-STAT3 and STAT3 were detected by Western blotting as previously described (Li et al., 2020). The primary antibodies were as follows: rabbit anti-phospho-STAT3 (1:2000, Cell Signaling Technology), mouse anti-STAT3 (1:1,000, Cell Signaling Technology) and mouse anti-β-actin (1:5,000, Sigma). Chemiluminescence was detected by ChemiDoc^TM^ Touch Imaging System (Bio-Rad).

### Primary Cultures and Oxygen-Glucose Deprivation

Brains of 18-day mouse embryos were used to prepare primary culture of cortical neurons, as previously described with modifications (Wang et al., 2016). Cerebral cortices were dissected in ice-cold Dulbecco’s modified Eagle’s medium (DMEM, Gibco) under a stereological microscope. Tissues were minced with scissors in ice-cold DMEM medium, and then filtered. Cells were plated at a density of 3.0×10^5^ cells/ml (for NKG2D detection) or 6.0×10^5^ cells/ml (for p-STAT3 detection) in DMEM supplemented with 10% fetal bovine serum (Gibco). After 6 h, the culture medium was replaced with Neurobasal Medium supplemented with 0.5 mM l-glutamine and 2% B27 serum-free supplement (Thermo Fisher Scientific).

For primary culture of glial cells, brains were removed from 24-h newborn mice and cerebral cortices were dissected in ice-cold HBSS (Hank’s Balanced Salt Solution). Tissues were mechanically dissociated and seeded in DMEM/F12 (Gibco) supplemented with 10% fetal bovine serum. Cells from one cortex were equally divided and seeded in three 35 mm culture dishes.

For OGD insult, cells were washed with D-Hanks (primary cortical neurons) or PBS (primary glial cells) and incubated in glucose-free DMEM (Gibco) in an anaerobic chamber (DG250, Whitley Workstation) filled with a mixture of 85% N_2_, 5% CO_2_ and 10% H_2_ at 37°C for 6 h. Then 1 g/L glucose was added, and the cells were returned to normoxic condition for 12 h. ASIV (50 μM) was added into the cultures 2 h before OGD and supplemented during OGD insult. Control cultures were maintained in DMEM for 18 h under normoxic condition. CT (10 μM) was added into the primary glial cell cultures 12 h before OGD to inhibit STAT3 phosphorylation.

### NK Cell Isolation and Purification

NK cells were isolated from the spleen using EasySep Mouse NK Cell Isolation Kit and purified using magnetic beads selection (STEMCELL Technologies) according to the manufacturer’s instructions, as previously described (Zhu et al., 2014). The purified NK cells were activated by the IL-2 (SL PHARM, 200 IU/ml) and LPS (Sigma, 5 μg/ml) at 37°C for 15 min.

### Calcein Release Assay

Calcein release assay was performed as previously described with modifications (Yao et al., 2018). Primary cortical neurons were subjected to 6-h OGD insult with or without ASIV treatment, and then the cells were returned to normoxic condition for 12 h. Cells were incubated with 2 μg/ml of calcein-AM (Thermo Fisher Scientific) at 37°C for 1 h with occasional shaking. Purified NK cells and OGD-treated neurons were mixed at an effector: target ratio of 5:1 and co-cultured in 96-well U-bottom plates for 4 h. Supernatants were transferred to a 96-well flat-bottom plate, and the fluorescence signal was detected using a Synergy 2 Multi-Mode Microplate Reader at 485 nm (for excitation) and 538 nm (for emission). The percentage of lysis was calculated according to a formula as follows: lysis % = [(experimental release - spontaneous release)/(maximum release - spontaneous release)] × 100.

### Migration Assay

Primary glial cells were seeded into the bottom chamber of a 24-well transwell plate with Matrigel Matrix-coated 3 μM pore size inserts (Corning) and subjected to OGD for 6 h with or without ASIV treatment. Then, purified NK cells (2×10^5^) were seeded into the upper chamber containing RPMI 1640 with 10% FBS at 37°C for 4 and 12 h. The fluid at 5 randomly selected fields in the lower chamber was collected in each well and the number of NK cells that migrated through the insert was counted under a phase contrast microscope (Leica, Germany).

### siRNA Transfection

The primary glial cells were transfected with 80 nM CCL2 or scrambled siRNA (GenePharma, Shanghai, China) using Lipofectamine 2000 (Invitrogen, MA, United States) according to the manufacturer’s instructions. The cells were used for subsequent experiments 48 h after transfection. The CCL2 siRNA sequences were 5′-GCU​AAU​GCA​UCC​ACU​ACC​UTT-3’ (forward) and 5′- AGG​UAG​UGG​AUG​CAU​UAG​CTT-3’ (reserve). The scrambled siRNA sequences were 5′-UUC​UCC​GAA​CGU​GUC​ACG​UTT-3’ (forward) and 5′-ACG​UGA​CAC​GUU​CGG​AGA​ATT-3’ (reverse).

### Statistical Analysis

Data are expressed as mean ± SD. Statistical analysis was performed using GraphPad Prism 8.0. Student’s t test and one-way ANOVA followed by Tukey post hoc test were used for comparisons between two and multiple groups, respectively. *p* < 0.05 was considered statistically significant.

## Results

### ASIV Attenuated Functional Deficits and Reduced Brain Infarction in MCAO Mice

**FIGURE 1:**
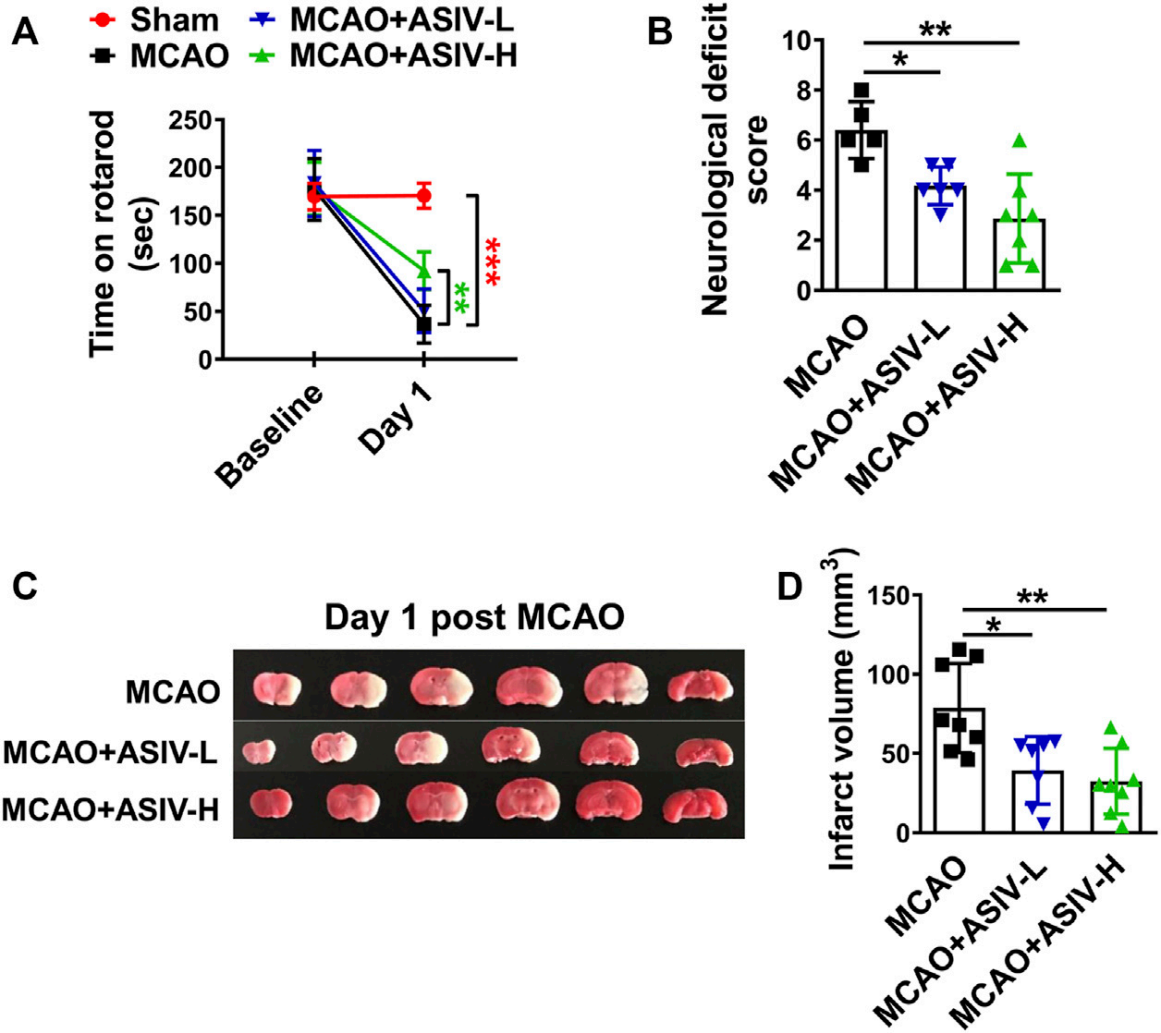
ASIV ameliorated functional deficits and reduced brain infarction in MCAO mice. **(A)** ASIV at 40 mg/kg significantly increased the rotarod retention time in MCAO mice on Day 1. **(B)** ASIV at both 20 and 40 mg/kg markedly reduced neurological deficit score of MCAO mice on Day 1. **(C,D)** ASIV at 40 mg/kg robustly reduced the brain infarct volume in MCAO mice on Day 1. ASIV-L: 20 mg/kg; ASIV-H: 40 mg/kg. *p < 0.05, **p < 0.01, ***p < 0.001; *n* = 6–8 per group.

We evaluated the protective effects of ASIV at 20 and 40 mg/kg in MCAO mice on Day 1 after reperfusion. As shown in Figure 1A, MCAO dramatically reduced the length of time that mice were able to stay on an accelerating rotarod, and ASIV at 40 mg/kg markedly increased the rotarod retention time from 36.5 ± 19.6 s to 92.3 ± 19.5 s. ASIV at 20 mg/kg did not improve the rotarod performance of MCAO mice. Severe neurological deficits had also been observed in MCAO mice, and these deficits were significantly attenuated by ASIV treatment at both 20 and 40 mg/kg (from 6.4 ± 1.1 to 4.2 ± 0.8 and 2.9 ± 1.8, respectively; Figure 1B). Furthermore, ASIV at 20 and 40 mg/kg robustly reduced brain infarct volume in MCAO mice from 78.7 ± 28.1 mm^3^ to 39.3 ± 21.2 mm^3^ and 32.4 ± 20.8 mm^3^, respectively (Figures 1C,D). Compared with 20 mg/kg, ASIV at 40 mg/kg provided better beneficial effects against brain ischemic injury. Therefore, 40 mg/kg was used in the subsequent studies.

### ASIV Inhibited Accumulation of NK Cells in the Ischemic Brain of MCAO Mice

**FIGURE 2:**
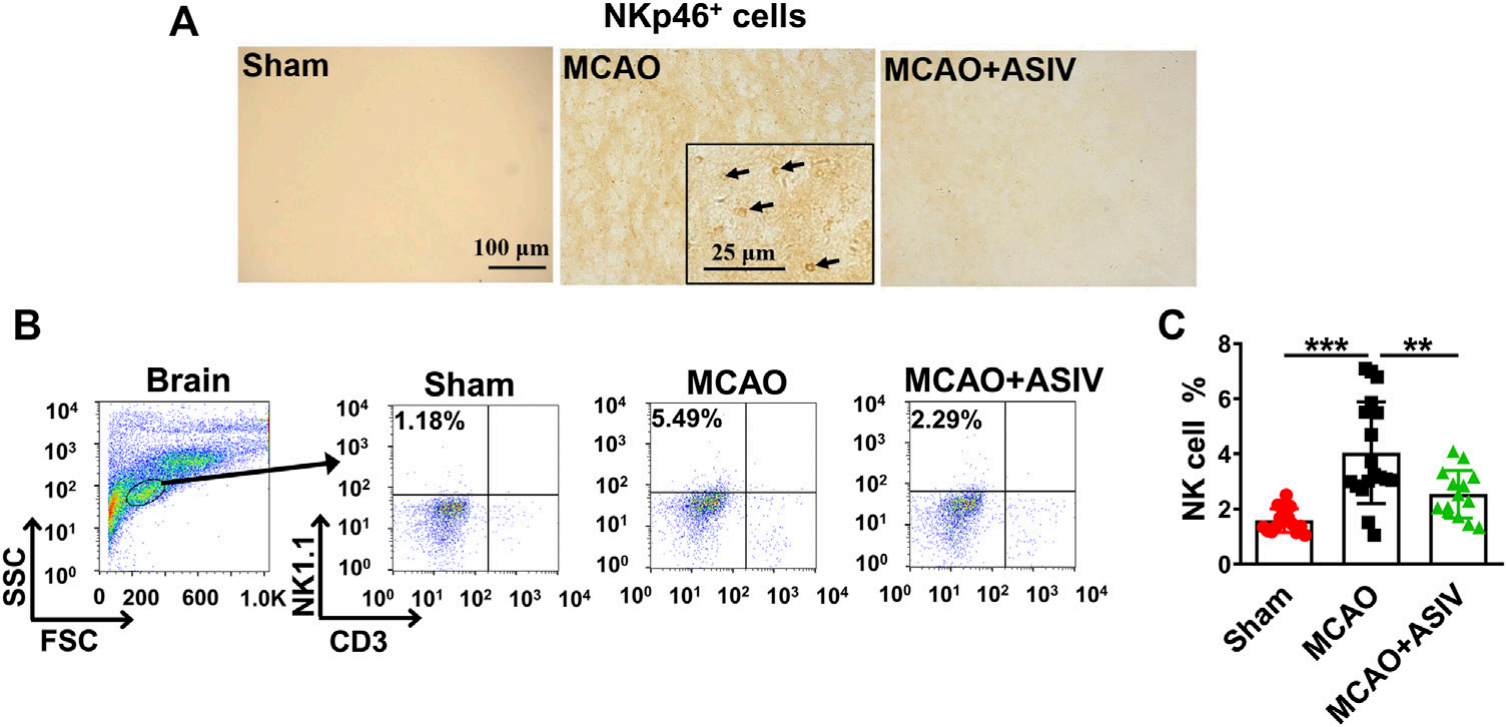
ASIV inhibited accumulation of NK cells in the ischemic brain of MCAO. **(A)** Abundant NKp46^+^ cells were observed in the ipsilateral cortex of MCAO mice on Day 1, as detected by immunohistochemistry. Accumulation of NKp46^+^ cells was nearly completely abrogated by ASIV at 40 mg/kg. **(B)** NK cells were identified as NK1.1^+^CD3^−^ lymphocytes using flow cytometry. **(C)** Approximately 4% of all the infiltrated lymphocytes in the ischemic hemisphere were NK cells in MCAO mice, and this percentage was reduced by half by ASIV treatment. **p < 0.01, ***p < 0.001; *n* = 14–17 per group.

NKp46 is a specific surface receptor expressed on human and murine NK cells (Walzer et al., 2007). There were abundant NKp46^+^ cells accumulating in the ischemic cortex on Day 1 after MCAO, and this accumulation was prevented by ASIV treatment (Figure 2A). Then, we performed flow cytometry to confirm the above findings. NK cells are CD3^−^ lymphocytes and NK1.1 is a NK cell specific marker in C57BL/6 mice (Hackett et al., 1986; Gan et al., 2014). Hence, NK cells were identified as NK1.1^+^CD3^−^ lymphocytes in flow cytometry (Figure 2B). Consistent with the above results, approximately 4% of all the infiltrated lymphocytes in the ischemic hemisphere were NK cells in MCAO mice, and this percentage was reduced by half by ASIV treatment (Figure 2C). These results clearly showed that ASIV inhibited NK cell brain infiltration in MCAO mice.

### Protection of ASIV Against Brain Ischemic Injury May Depend on NK Cell Inhibition

**FIGURE 3:**
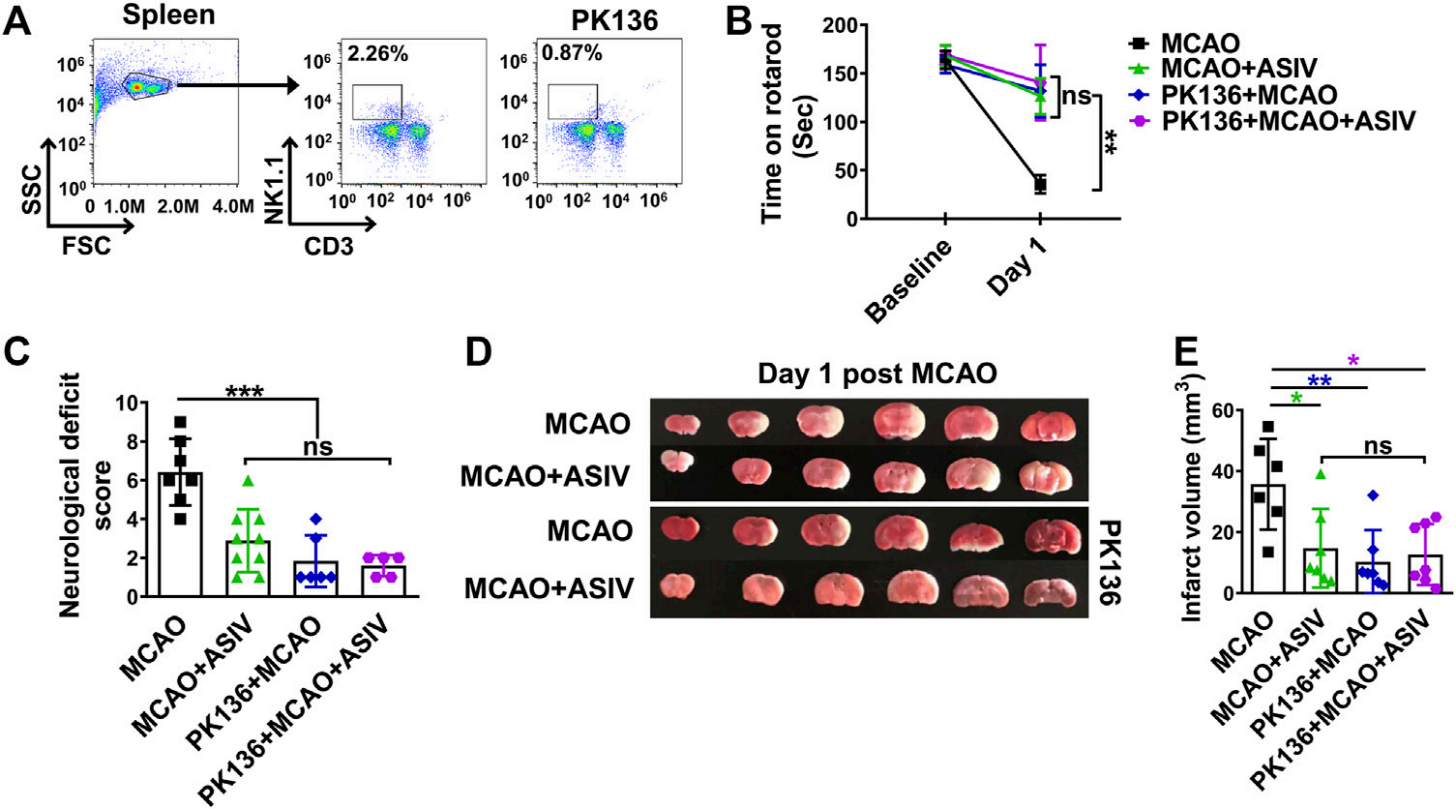
Beneficial effects of ASIV against brain ischemic injury may depend on NK cell inhibition. **(A)** PK136, an anti-NK1.1 monoclonal antibody, nearly completely depleted NK cells in the spleen. MCAO mice treated with ASIV, PK136 or both spent much longer time on an accelerating rotarod **(B)**, had less neurological deficits score **(C)** and had smaller brain infarct volume **(D,E)**, compared with vehicle-treated MCAO group. For the above parameters, there was no statistical difference among these three treatment groups. *p < 0.05, **p < 0.01, ***p < 0.001; ns, non-statistical significance. *n* = 6–9 per group.

The aforementioned results showed that ASIV protected brain ischemic injury and inhibited NK cell post-ischemic brain infiltration. These findings raised the possibility that the ability of inhibiting NK cells may contribute to the overall beneficial effects of ASIV on brain ischemic injury. To address this possibility, we investigated the effects of ASIV on post-ischemic functional deficits and brain infarction after NK cells were depleted by PK136, an anti-NK1.1 monoclonal antibody. As shown in Figure 3A, PK136 nearly completely depleted NK cells in the spleen. NK cell depletion markedly improved the rotarod performance, attenuated neurological deficits and diminished brain infarction in MCAO mice (Figures 3B–E). These results confirmed the detrimental role of NK cells in the acute phase of brain ischemia. Moreover, ASIV did not further enhance the beneficial effects of NK cell depletion on functional deficits and brain infarction in MCAO mice. There was no significant difference among MCAO + ASIV, PK136 + MCAO, and PK136 + MCAO + ASIV groups (Figures 3B–E). These results strongly suggested that the protective effects of ASIV on brain ischemic injury may depend on NK cell inhibition.

### ASIV Inhibited Post-ischemic NK Cell Brain Infiltration Through Suppressing Glial Cell-Derived CCL2-Mediated NK Cell Migration

**FIGURE 4:**
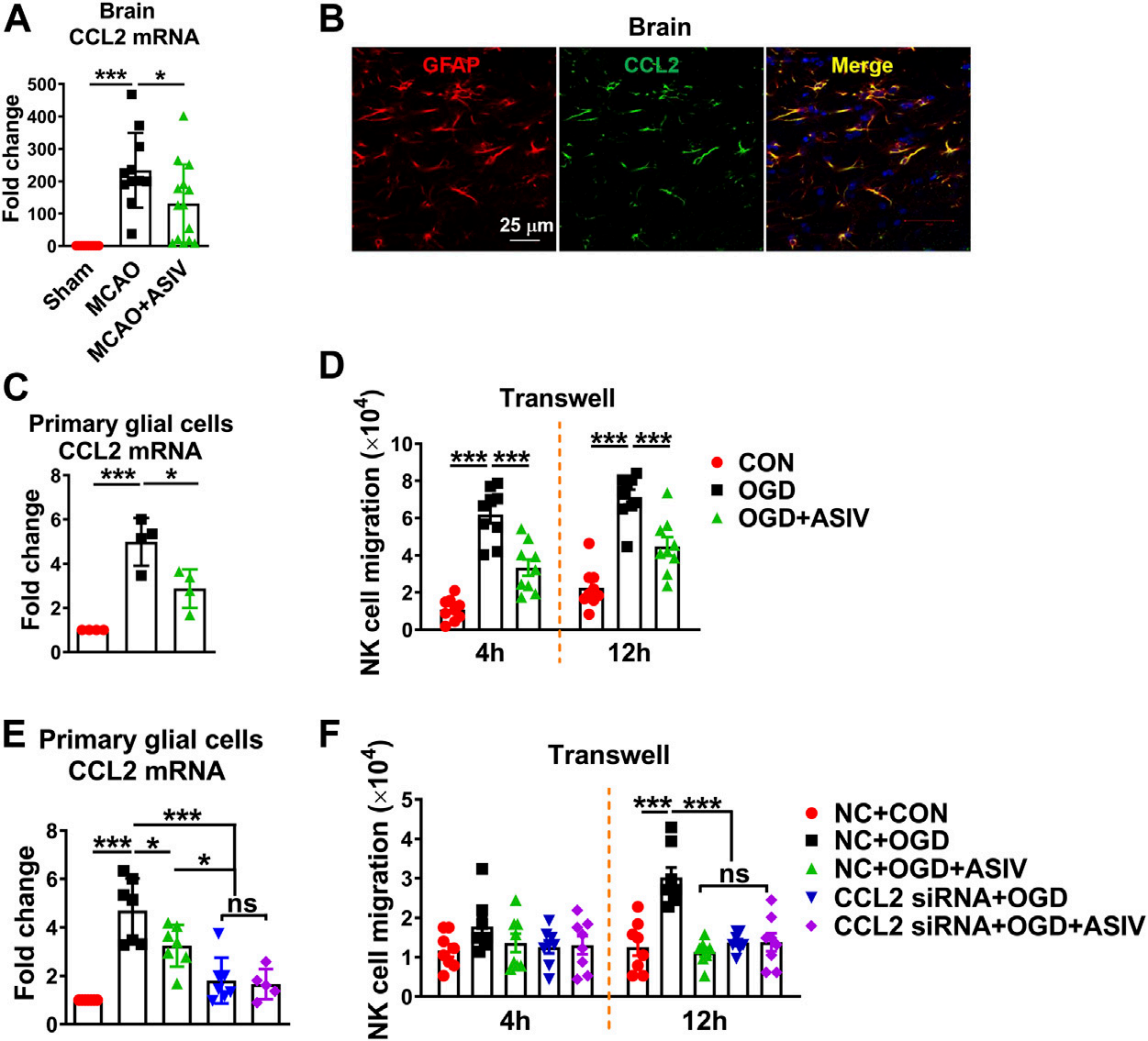
ASIV inhibited post-ischemic brain infiltration of NK cells through suppressing ischemic glial cell-derived CCL2-mediated NK cell migration. **(A)** CCL2 mRNA level was dramatically increased in the ischemic cortex and was robustly reduced by ASIV treatment. **(B)** CCL2 was co-localized with astrocytes as labeled with GFAP in the ischemic cortex. **(C)** ASIV markedly reduced OGD-induced upregulation of CCL2 mRNA in primary cortical glial cells. **(D)** NK cells were co-cultured with OGD-treated glial cells in a transwell chamber. After 4 and 12 h, a number of NK cells migrated towards OGD-treated glial cells, and the migration was significantly suppressed by ASIV. **(E)** CCL2 mRNA expression was robustly knocked down by siRNA in OGD-treated glial cells. **(F)** NK cell migration towards OGD-treated glial cells was dramatically suppressed when CCL2 was knocked down. In addition, ASIV did not enhance the inhibitory effect of CCL2 siRNA on NK cell migration. *p < 0.05, ***p < 0.001; ns, non-statistical significance. For *in vivo* studies, *n* = 11–14 per group. For *in vitro* studies, experiments were repeated 3 times.

Among all MCAO-upregulated chemokines, CCL2 was the most significantly upregulated one in the ischemic brain on Day 1 after MCAO (data not shown). Compared with sham controls, CCL2 mRNA level increased 234-fold in the ischemic cortex and ASIV decreased it to 131-fold (Figure 4A). As revealed by immunofluorescence staining, CCL2 was well co-localized with GFAP^+^ cells, suggesting that CCL2 was mainly expressed by astrocytes in the ischemic brain (Figure 4B). However, CCL2 mRNA expression was mildly increased in mouse primary cortical astrocytes subjected to OGD insult (data not shown). We observed a marked increase in CCL2 mRNA level in OGD-treated mixed primary cortical glial cells. These results suggested that the interaction between astrocytes and microglia may be required for astrocytes to produce CCL2 under ischemic insult. Therefore, mixed glial cells were used in the subsequent *in vitro* experiments. Consistent with the *in vivo* results, we found that the mRNA level of CCL2 increased 5-fold in OGD-treated glial cells and ASIV reduced it to 2.9-fold compared with control cells (Figure 4C).

To investigate whether glial cells can induce NK cell migration under ischemic condition and whether ASIV can affect the migration, primary mouse cortical glial cells were subject to OGD insult with or without ASIV treatment and then co-cultured with NK cells using the transwell system. As shown in Figures 4D, a number of NK cells migrated towards OGD-treated glial cells at 4 and 12 h after co-culture, and the migration of NK cells was markedly suppressed by ASIV at both time points. In addition, we used siRNA to block CCL2 expression in the primary glial cells and confirmed that CCL2 mRNA expression was robustly suppressed in the OGD-treated glial cells (Figure 4E). ASIV treatment did not enhance the effect of CCL2 siRNA to suppress CCL2 mRNA expression. After CCL2 was knocked down, OGD-treated glial cells failed to attract NK cell migration in the transwell system at 12 h after co-culture, suggesting that NK cell migration induced by ischemic glial cells was CCL2-dependent (Figure 4F). Furthermore, ASIV did not further suppress NK cell migration toward OGD-treated glial cells when CCL2 was knocked down. There was no significant difference in NK cell migration among OGD + ASIV, OGD + CCL2 siRNA and OGD + CCL2 siRNA + ASIV groups (Figure 4F). Together, these findings showed that ASIV suppressed NK cell brain infiltration through inhibiting glial cell-derived CCL2 in the ischemic brain.

### ASIV Abrogated NK Cell-Mediated Cytolytic Killing of Ischemic Neurons and Abolished NK Cell-Derived IFN-γ Production in the Ischemic Brain

**FIGURE 5:**
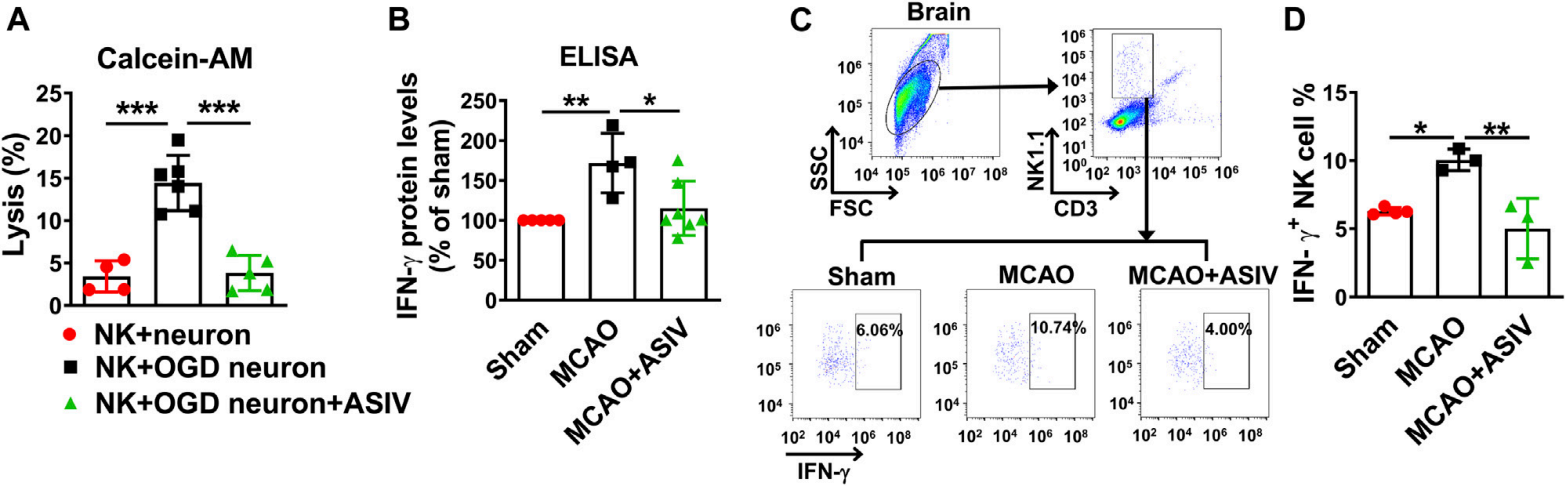
ASIV prevented NK cell-mediated cytolytic killing of ischemic neurons and abolished NK cell-derived IFN-γ expression in the ischemic brain **(A)** NK cell-mediated cytolytic killing of neurons was evaluated by calcein release assay. After co-cultured for 4 h, ASIV nearly completely abolished the NK cell-induced killing of OGD-treated primary cortical neurons. **(B)** As measured by ELISA, IFN-γ protein level was significantly increased in the ischemic brain, whereas ASIV treatment robustly reduced the upregulation of IFN-γ. **(C,D)** As assessed by flow cytometry, ASIV completely inhibited the upregulation of NK cell-derived IFN-γ in the ischemic brain. *p < 0.05, **p < 0.01, ***p < 0.001. For *in vivo* studies, *n* = 3–7 per group. For *in vitro* studies, experiments were repeated 3 times.

The effect of ASIV on NK cell-mediated cytolytic killing of ischemic neurons was evaluated by calcein release assay. NK cells induced severe killing of OGD-treated primary cortical neurons after co-culture for 4 h, and the killing was nearly completely abrogated by ASIV treatment (Figure 5A).

In addition to cytolytic effects, activated NK cells are a major source of IFN-γ which can boost inflammation. As assessed by ELISA, we found the protein level of IFN-γ in the ischemic cortex was significantly upregulated on Day 1 after MCAO mice and ASIV treatment markedly reduced the upregulation of IFN-γ (Figure 5B). We further used flow cytometry to investigate the effect of ASIV on IFN-γ secreted by brain-infiltrated NK cells. In line with the above result, approximately 10% infiltrated NK cells produced IFN-γ in the ischemic hemisphere of MCAO mice, compared with 6% in sham-operated group (Figures 5C,D). ASIV robustly reduced the ratio of IFN-γ-secreting NK cells in the ischemic hemisphere of MCAO mice to that in sham-operated mice.

### ASIV Suppressed Infiltrated NK Cell-Expressed NKG2D in the Ischemic Brain

**FIGURE 6:**
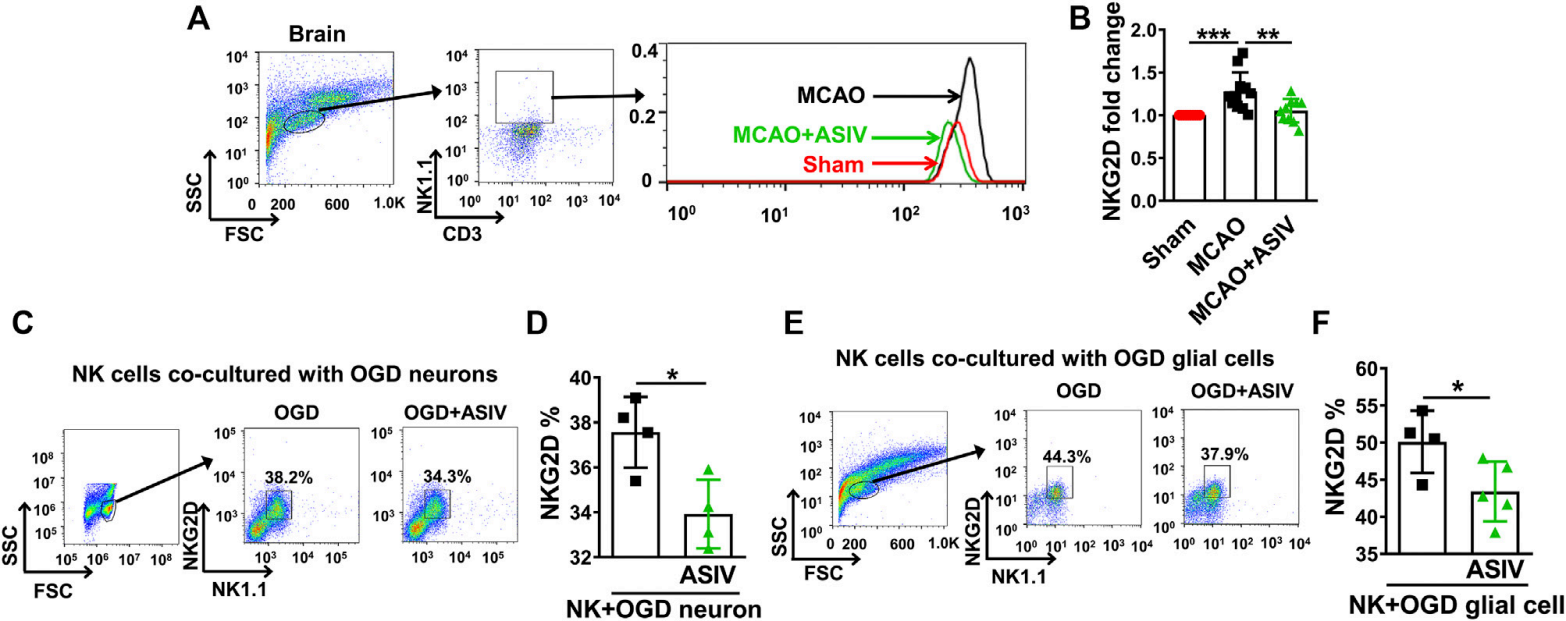
ASIV suppressed infiltrated NK cell-expressed NKG2D in the ischemic brain. **(A,B)** As assessed by flow cytometry, the expression of NKG2D on the surface of NK cells was significantly increased in the ischemic brain, which was robustly downregulated by ASIV treatment. *In vitro*, ASIV also significantly suppressed the upregulated NKG2D expression on the surface of NK cells co-cultured with OGD-treated primary neurons **(C,D)** or glial cells **(E,F)**. *p < 0.05, **p < 0.01, ***p < 0.001. For A and B, *n* = 10–11 per group. For C to F, experiments were repeated 3 times.

We studied whether ASIV could affect the levels of NKG2D, a key activating receptor on NK cells, in the ischemic brain on Day 1 after MCAO. Expression of NKG2D on the surface of brain-infiltrated NK cells was evaluated using flow cytometry. On Day 1 after MCAO, NKG2D expression was significantly increased on the surface of brain-infiltrated NK cells in the ischemic hemisphere (1.3-fold) compared with that in sham-operated group (the MFI was 64.1 ± 18.9 for sham and 82.4 ± 21.6 for MCAO), and this upregulation was markedly suppressed by ASIV treatment (1.1-fold, the MFI was 69.0 ± 19.8, Figures 6A,B). In addition, we investigated the effect of ASIV on NKG2D level on the surface of NK cells co-cultured with OGD-treated primary cortical neurons or glial cells. Primary neurons or glial cells were subjected to 6-h OGD and then co-cultured with NK cells for 12 h with or without ASIV treatment. As shown in Figures 6C–F, ASIV significantly reduced NKG2D level on the surface of NK cells co-cultured with primary neurons and glial cells subjected to OGD insult, respectively. Together with the data in Figure 5, our results indicated that ASIV suppressed the activation of brain-infiltrated NK cells in MCAO mice.

### ASIV Inhibited CCL2-Mediated NK Cell Brain Infiltration by Suppressing STAT3 Activation in the Ischemic Brain

**FIGURE 7:**
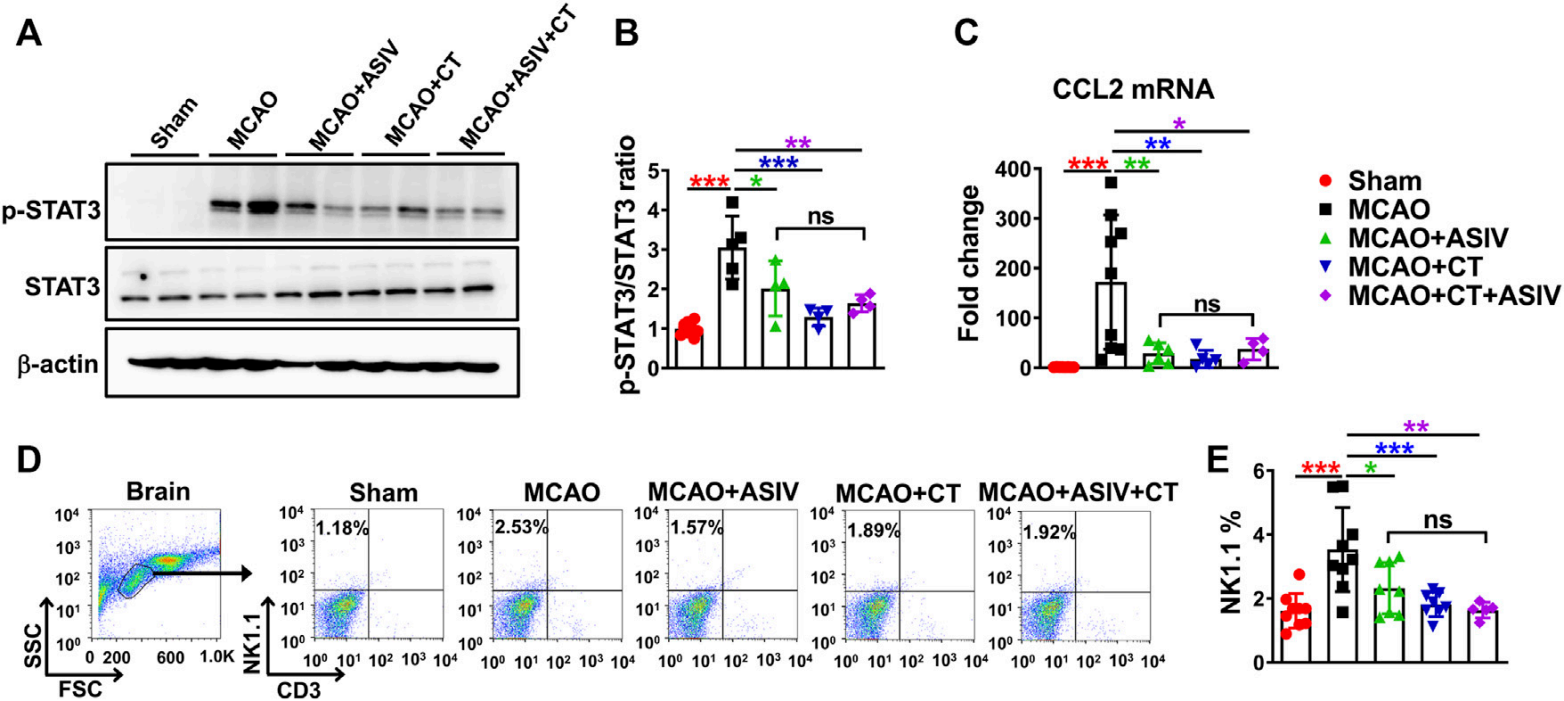
ASIV inhibited CCL2-mediated NK cell brain infiltration by suppressing STAT3 activation in the ischemic brain. Both ASIV and CT, a STAT3 inhibitor, significantly inhibited the upregulation of phosphorylated STAT3 at Tyr705 **(A,B)**, CCL2 mRNA level **(C)** and NK cell brain infiltration **(D,E)** in the ischemic hemisphere. Notably, there was no additive or synergistic effect on STAT3 phosphorylation, CCL2 mRNA level and NK cell brain infiltration when ASIV and CT were used together. *p < 0.05, **p < 0.01, ***p < 0.001; ns, non-statistical significance. *n* = 4–9 per group.

STAT3, a key transcriptional factor involved in inflammation and immunity, can facilitate the expression of chemokines, including CCL2. On Day 1 after MCAO, compared with that in sham-operated mice, Tyr705 phosphorylation of STAT3 was increased to 3-fold in the ischemic cortex of MCAO mice, and this increase was robustly suppressed by ASIV (2-fold) and CT (1.3-fold), a STAT3 inhibitor, respectively (Figures 7A,B). As expected, CT also significantly reduced MCAO-induced upregulation of CCL2 mRNA level (from 172-fold to 17-fold, compared with sham-operated mice, Figure 7C). Moreover, ASIV did not enhance the inhibitory effect of CT on STAT3 phosphorylation or CCL2 mRNA expression in the ischemic cortex of MCAO mice. There was no significant difference in STAT3 phosphorylation or CCL2 mRNA expression among MCAO + ASIV, MCAO + CT and MCAO + ASIV + CT groups (Figures 7A–C). Furthermore, the inhibitory effect of ASIV on NK cell brain infiltration was mimicked by CT. As a result of STAT3 inhibition and CCL2 downregulation, CT significantly inhibited NK cell infiltration in the ischemic hemisphere (from 3.53 to 1.82%). Of note, there was no additive or synergistic effect on NK cell brain infiltration when ASIV and CT were used together (Figures 7D,E). Our findings suggested that ASIV inhibited CCL2-mediated NK cell brain infiltration by suppressing STAT3 activation in the ischemic brain.

### ASIV Inhibited Activation of Brain-Infiltrated NK Cells by Suppressing STAT3 Activation

**FIGURE 8:**
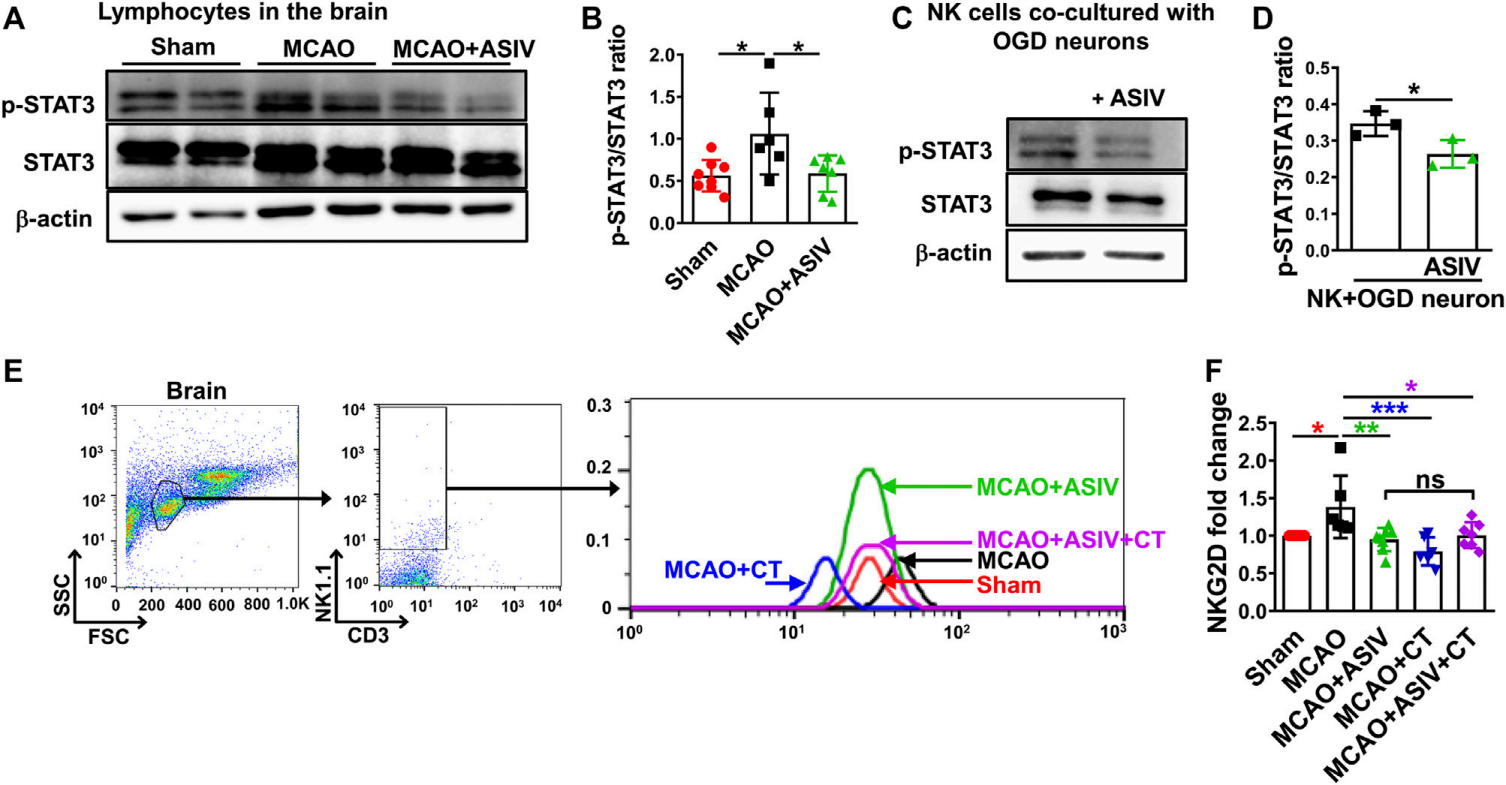
ASIV inhibited activation of brain-infiltrated NK cells by suppressing STAT3 activation. **(A,B)** The phosphorylated STAT3 at Tyr705 was increased in the lymphocytes of ischemic brain, and this increase was suppressed by ASIV treatment. **(C,D)** ASIV also markedly reduced the phosphorylated STAT3 in NK cells co-cultured with OGD-treated neurons. **(E,F)** Both ASIV and CT significantly reduced NKG2D upregulation on the surface of infiltrated NK cells in the ischemic hemisphere. There was no additive or synergistic effect on NKG2D expression on brain-infiltrated NK cells when ASIV and CT were used together. *p < 0.05, **p < 0.01, ***p < 0.001; ns, non-statistical significance. For *in vivo* studies, *n* = 6–8 per group. For *in vitro* studies, experiments were repeated 3 times.

NK cell function, including transcription of NKG2D and IFN-γ release, has been shown to be regulated by STAT3 activation (Zhu et al., 2014; Jin et al., 2018). We found that STAT3 phosphorylation at Tyr705 was significantly increased in the lymphocytes isolated from the ischemic hemisphere in MCAO mice and this increase was robustly reversed by ASIV treatment (Figures 8A,B). In addition, ASIV markedly reduced STAT3 phosphorylation in NK cells co-cultured with OGD-treated primary cortical neurons (Figures 8C,D). Furthermore, STAT3 inhibition induced by CT significantly reduced NKG2D expression on brain-infiltrated NK cells and ASIV did not enhance the effect of CT on NKG2D downregultion (Figures 8E,F). There was no significant difference in NKG2D expression among MCAO + ASIV, MCAO + CT and MCAO + ASIV + CT groups. The MFI was 50.8 ± 20.1 for sham, 72.2 ± 32.2 for MCAO, 50.7 ± 24.2 for MCAO + ASIV, 41.2 ± 20.8 for MCAO + CT, and 58.6 ± 20.2 for MCAO + ASIV + CT. These results suggested that ASIV inhibited activation of brain-infiltrated NK cells by suppressing STAT3 activation.

## Discussion

Accumulating evidence indicates that immune responses may shape the outcome of stroke. Due to unique immunology features and the detrimental role during the acute phase of ischemic stroke, NK cells are receiving growing attentions as a promising target for immune interventions in treatment of ischemic stroke (Fu et al., 2015). In this study, we found that ASIV inhibited NK cell brain infiltration during the acute stage of brain ischemia. Concurrently, ASIV suppressed activation of these infiltrated NK cells through inhibiting direct cytolytic killing of ischemic neurons and suppressing their IFN-γ secretion and NKG2D expression in the ischemic brain. Furthermore, the inhibitory effect of ASIV on inhibiting post-ischemic brain infiltration and activation of NK cells may be attributed to STAT3 inhibition. The immunoregulatory ability of ASIV on NK cells may eventually contribute to its protective effects against acute brain ischemic injury in MCAO mice.

Accumulation of NK cells has been observed in the ischemic brain of both patients and rodents at the acute stage of ischemic stroke (Gan et al., 2014; Zhang et al., 2014; Dou et al., 2018). Research in rodents has revealed the detrimental effects of brain-infiltrated NK cells (Gan et al., 2014; Zhang et al., 2014). In this study, we found that NK cell depletion using anti-NK1.1 antibody provided protective effects against brain ischemic injury, including reducing brain infarction, prolonging rotarod retention time and ameliorating neurological deficits in MCAO mice. These findings confirmed the detrimental role of NK cells in ischemic brain injury. Notably, ASIV did not further enhance the protective effects of NK cell depletion against brain infarction and neurological deficits. In terms of the protection against brain ischemic injury, there was no significant difference among ASIV, NK depletion and ASIV + NK depletion groups. Hence, the beneficial effects of ASIV against brain ischemic injury may depend on its inhibition of NK cells.

Immune cell migration depends on chemotaxis. CCL2 is one of the main chemokines which drive migration of immune cells into the ischemic brain parenchyma (Bose and Cho, 2013). In rodents, CCL2 is upregulated in the ischemic brain within hours after ischemic onset and the upregulation lasts for days (Wang et al., 1995; Che et al., 2001). At 24 h after ischemic onset, increased CCL2 expression have also been observed in both the cerebrospinal fluid and serum of ischemic stroke patients (Losy and Zaremba, 2001; Arakelyan et al., 2005). During the early phase of ischemic stroke, the prompt upregulation of CCL2 suggests its key role in recruiting innate immune cells, such as NK cells. In support of this notion, CCL2 deficiency results in less infiltration of immune cells, smaller brain infarction, and reduced BBB leakage in MCAO mice (Hughes et al., 2002; Strecker et al., 2013). In this study, we found that CCL2, mainly derived from astrocytes, was dramatically increased in the ischemic cortex on Day 1 after MCAO and ASIV robustly inhibited the CCL2 upregulation. Our results also implied that microglia may be required for astrocytes to induce CCL2 under ischemic insult. Hence, we used mixed primary cortical glial cells in the subsequent *in vitro* experiments. As a result of CCL2 upregulation, OGD-treated glial cells induced migration of NK cells in a transwell system. This migration was robustly suppressed by ASIV and CCL2 siRNA, respectively. Notably, ASIV did not enhance the inhibitory effect of CCL2 knockdown on NK cell migration towards OGD-treated glial cells. Together, our findings indicate that ASIV may attenuate post-ischemic NK cell brain infiltration via inhibiting glial cell-derived CCL2.

NK cells can direct kill ischemic neurons through cytolytic effects (Gan et al., 2014). In line with this notion, we found that NK cell-mediated killing of OGD-treated primary cortical neurons was nearly completely blocked by ASIV. In addition to direct killing of ischemic neurons, NK cells can release IFN-γ which can exacerbate stroke outcomes in mouse MCAO models (Yilmaz et al., 2006; Gan et al., 2014). IFN-γ-deficient NK cells have been shown to reduce brain lesions and attenuate neurological deficits in MCAO mice (Gan et al., 2014). We showed that ASIV completely suppressed IFN-γ expression released from infiltrated NK cells in the ischemic brain, suggesting that suppression of NK cell-derived IFN-γ contributed to the inhibitory effects of ASIV on NK cell activation.

NKG2D is a major activating receptor of NK cells and can activate NK cell cytolytic responses (Bauer et al., 1999). In the ischemic mouse brain, upregulation of NKG2D on infiltrated NK cells has been shown to contribute to the loss of NK cell tolerance in ischemic neurons (Gan et al., 2014). After brain ischemia, we found that NKG2D expression on brain-infiltrated NK cells was increased. Strikingly, ASIV inhibited NKG2D expression on these NK cells in the ischemic brain. Furthermore, ASIV also significantly reduced the NKG2D expression on NK cells co-cultured with OGD-treated primary cortical neurons or glial cells. These *in vitro* results confirm the inhibitory effects of ASIV on NKG2D expression on brain-infiltrated NK cells under ischemic condition. Together, our findings suggest that ASIV can inhibit activation of brain-infiltrated NK cells through preventing their direct cytolytic killing of ischemic neurons, abrogating their IFN-γ production, and suppressing their NKG2D expression in the ischemic brain.

STAT signaling has been identified as a key transcription factor controlling immunity (Shuai and Liu, 2003). STAT3 is involved in regulating the expression of chemokines, including CCL2, which contribute to the recruitment of immune cells (Fletcher et al., 2019). In support of this notion, we found that STAT3 inhibition markedly suppressed CCL2 expression in the ischemic cortex, and subsequently inhibited the brain-infiltration of NK cells in MCAO mice. These effects of STAT3 inhibition echoed those of ASIV. In the meantime, STAT3 also controls NK cell activation. NKG2D expression in NK cells can be regulated by STAT3 at the transcriptional level. NKG2D expression is decreased in NK cells isolated from STAT3 knockout mice, and human NK cells with dominant-negative STAT3 mutations have blunted NKG2D responses to STAT3-activating cytokines (Zhu et al., 2014). Further study indicates the direct transcriptional regulation of NKG2D by phosphorylated STAT3 at Tyr 705, and inhibition of STAT3 tyrosine phosphorylation can suppress NKG2D expression in NK cells (Zhu et al., 2014; Ni et al., 2017). In addition, STAT3 activation has also been shown to preserve NK cell function by increasing IFN-γ release in the splenic NK cells after brain ischemia (Jin et al., 2018). Consistent with the above findings, STAT3 phosphorylation at Tyr705 was markedly increased both in the infiltrated lymphocytes in the ischemic cortex and in NK cells co-cultured with OGD-treated neurons, and ASIV robustly inhibited STAT3 phosphorylation both *in vivo* and *in vitro*. Furthermore, inhibition of STAT3 significantly reduced NKG2D expression on the surface of infiltrated NK cells in the ischemic hemisphere, which mimicked the effect of ASIV. Notably, ASIV did not enhance the effect of STAT3 inhibition on CCL2-mediated NK cell brain-infiltration and NKG2D expression in these NK cells in the ischemic brain, suggesting that the effects of ASIV on inhibiting post-ischemic brain-infiltration and activation of NK cells may be attributed to STAT3 suppression.

In conclusion, ASIV inhibited the brain infiltration and activation of NK cells during the acute stage of brain ischemia in mice, and these effects of ASIV likely depended on STAT3 inhibition. The inhibition of NK cells eventually contributed to the beneficial effects of ASIV against acute brain ischemic injury. Our findings suggest that ASIV is a promising therapeutic candidate in NK cell-based immunotherapy for the treatment of acute ischemic stroke and pave the way for potential clinical trials.

## Data Availability

The raw data supporting the conclusion of this article are available in the Supplementary Material.
