# Supplementary figures and images for: Suppressing NK Cells by Astragaloside IV Protects Against Acute Ischemic Stroke in Mice Via Inhibiting STAT3
Source: Front Pharmacol. 2022 Feb 3;12:802047. doi: 10.3389/fphar.2021.802047 (PMC8852846; doi:10.3389/fphar.2021.802047)

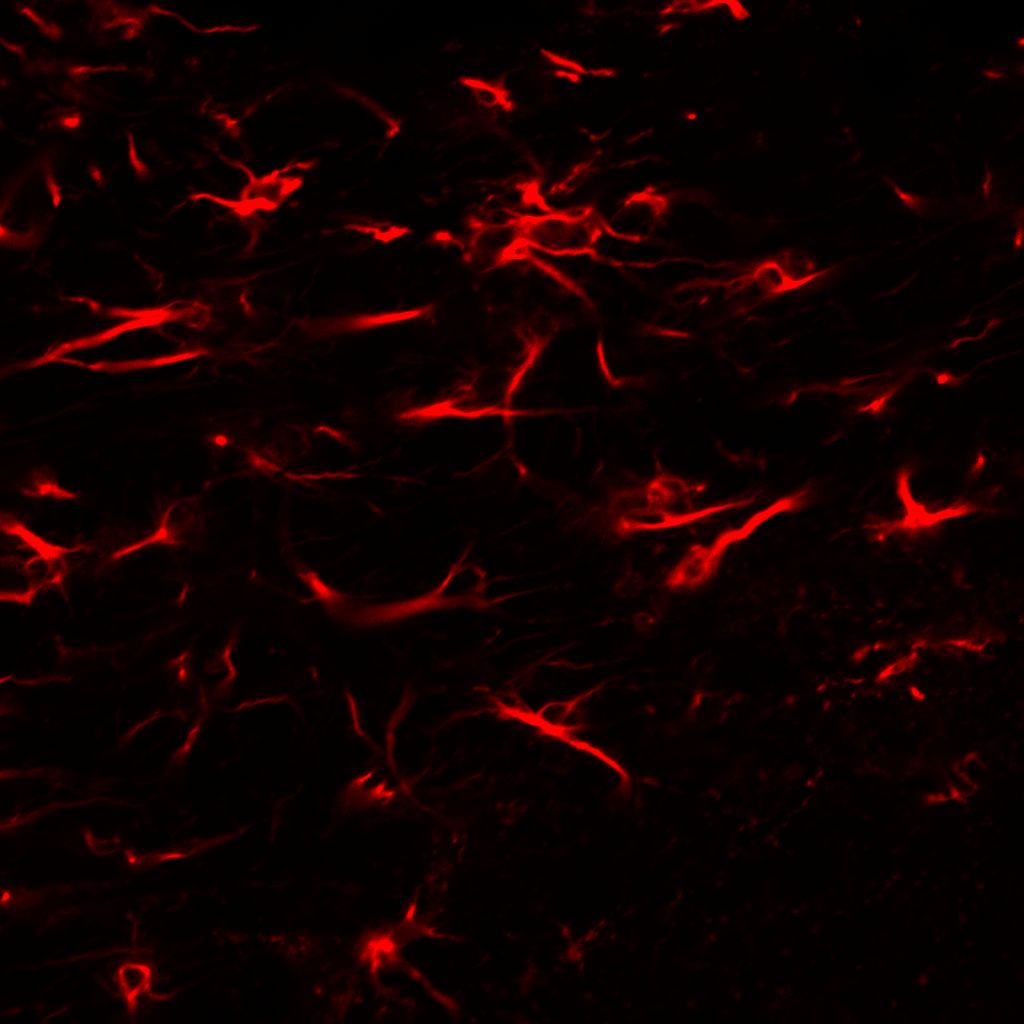

Supplement: Supplementary file 1 [file Image6.TIF]

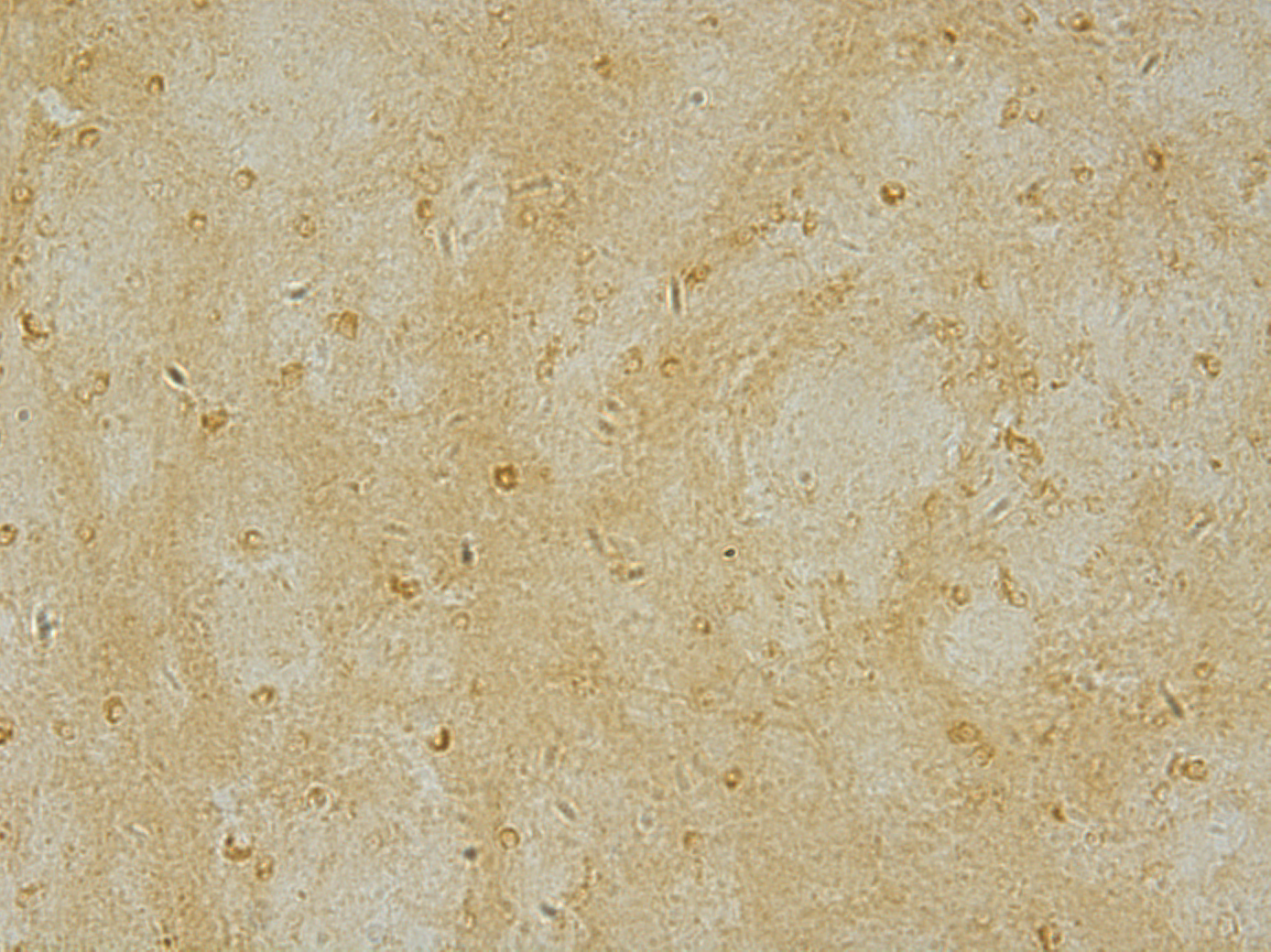

Supplement: Supplementary file 2 [file Image3.TIF]

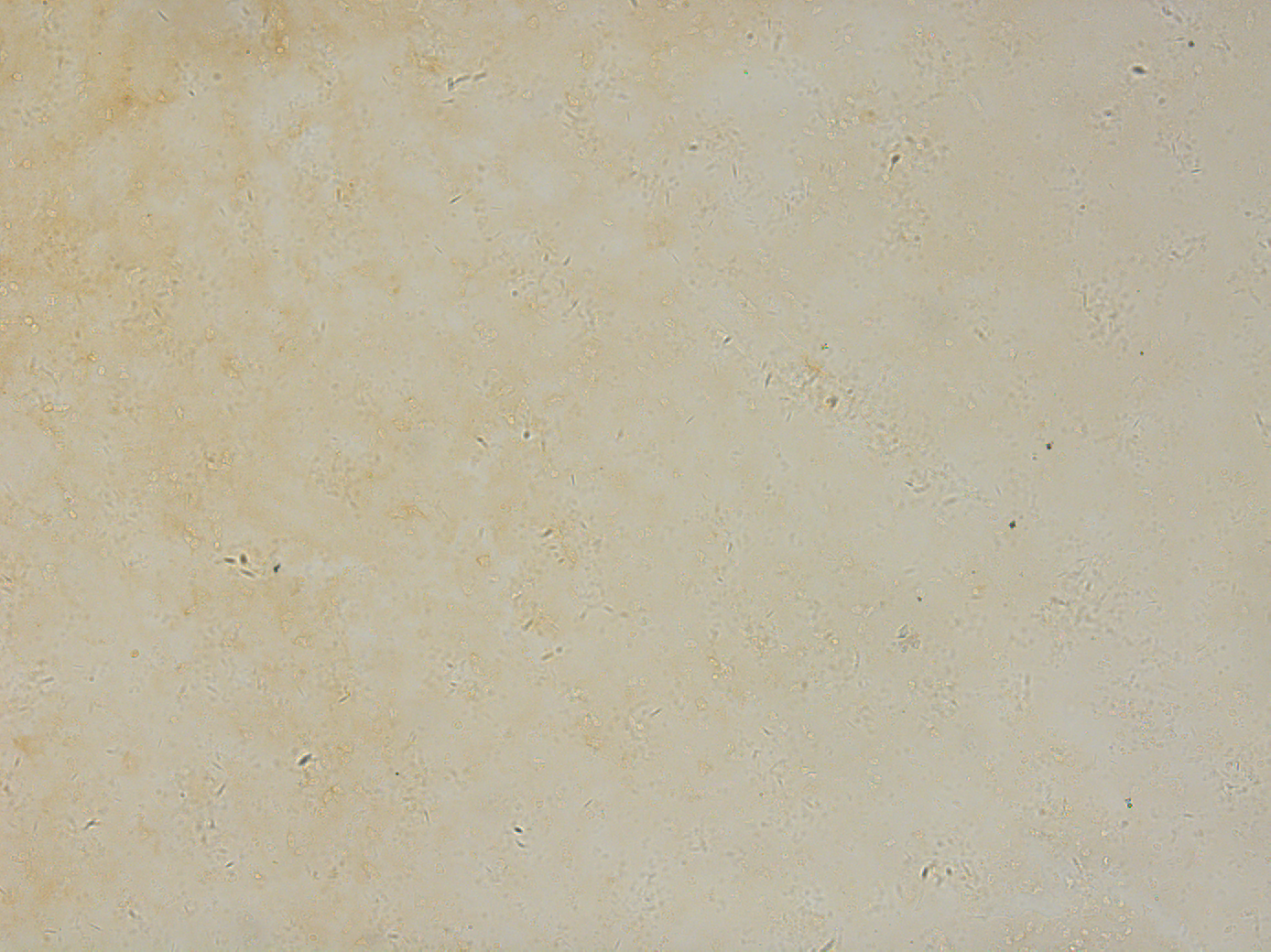

Supplement: Supplementary file 3 [file Image4.TIF]

**Figure 7A:**

**
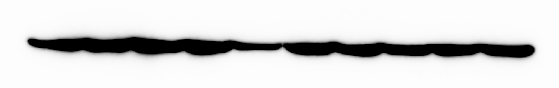
β-actin**

**
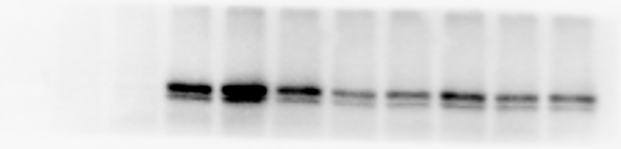
**

**p-STAT3**

**
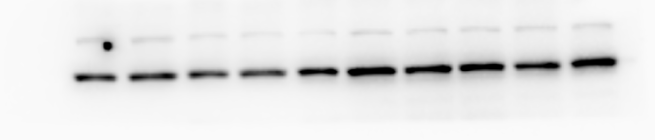
STAT3**

**Figure 8A:**

**
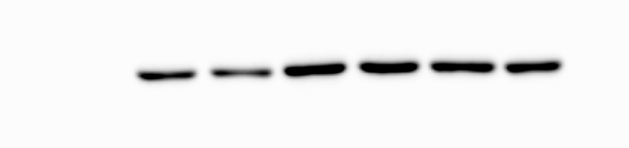
**

**β-actin**

**
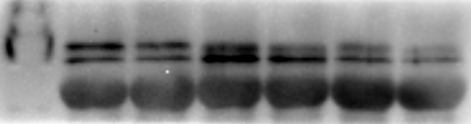
**

**p-STAT3**

**
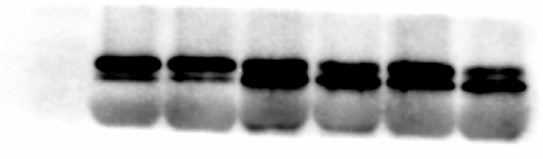
**

**STAT3**

**Figure 8C:**

**
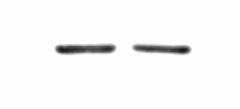
**

**β-actin**

**
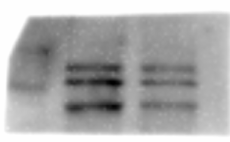
**

**p-STAT3**

**
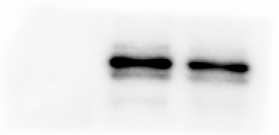
**

**STAT3**

Supplement: Supplementary file 4 [file DataSheet3.docx]

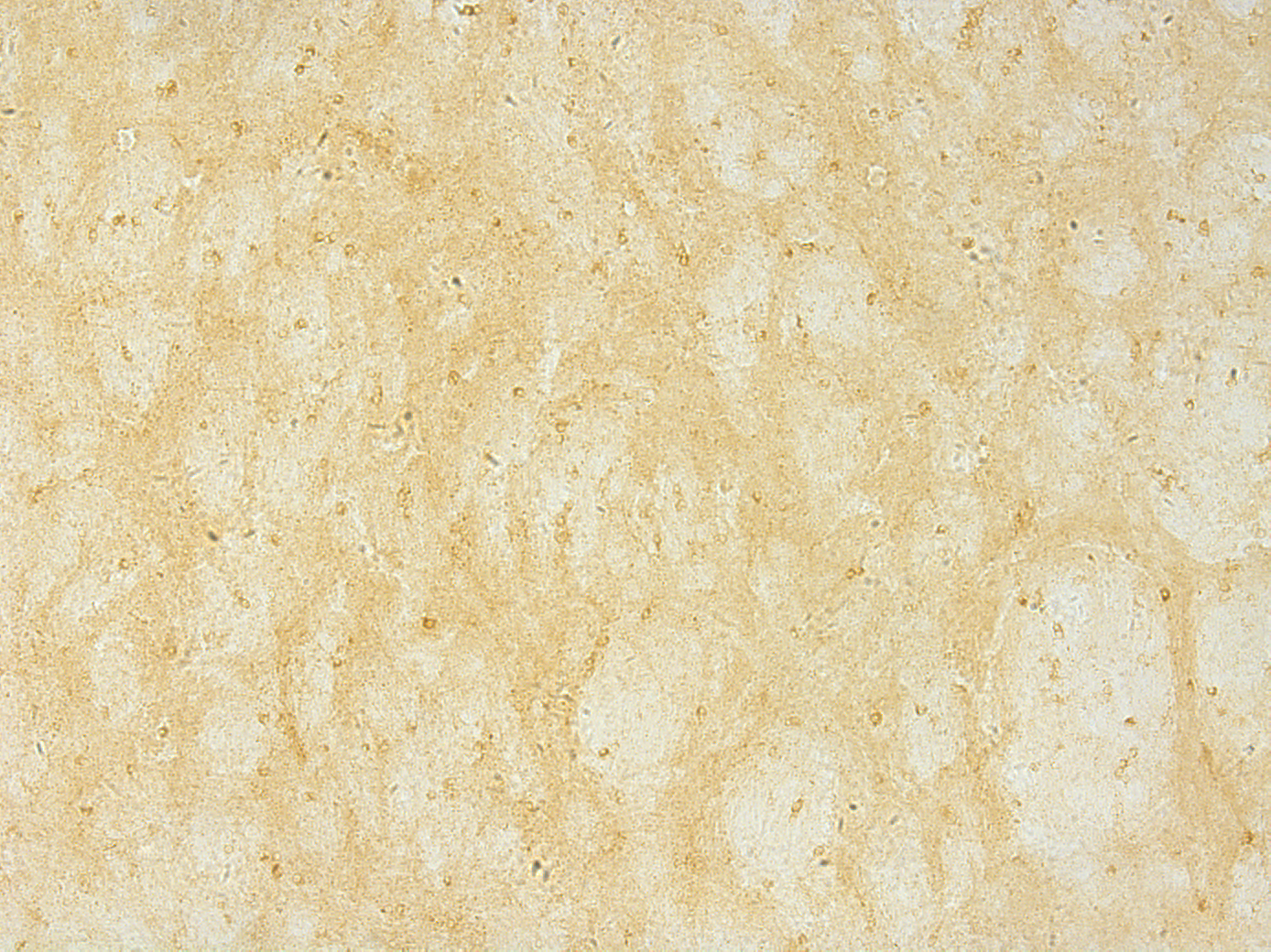

Supplement: Supplementary file 5 [file Image2.TIF]

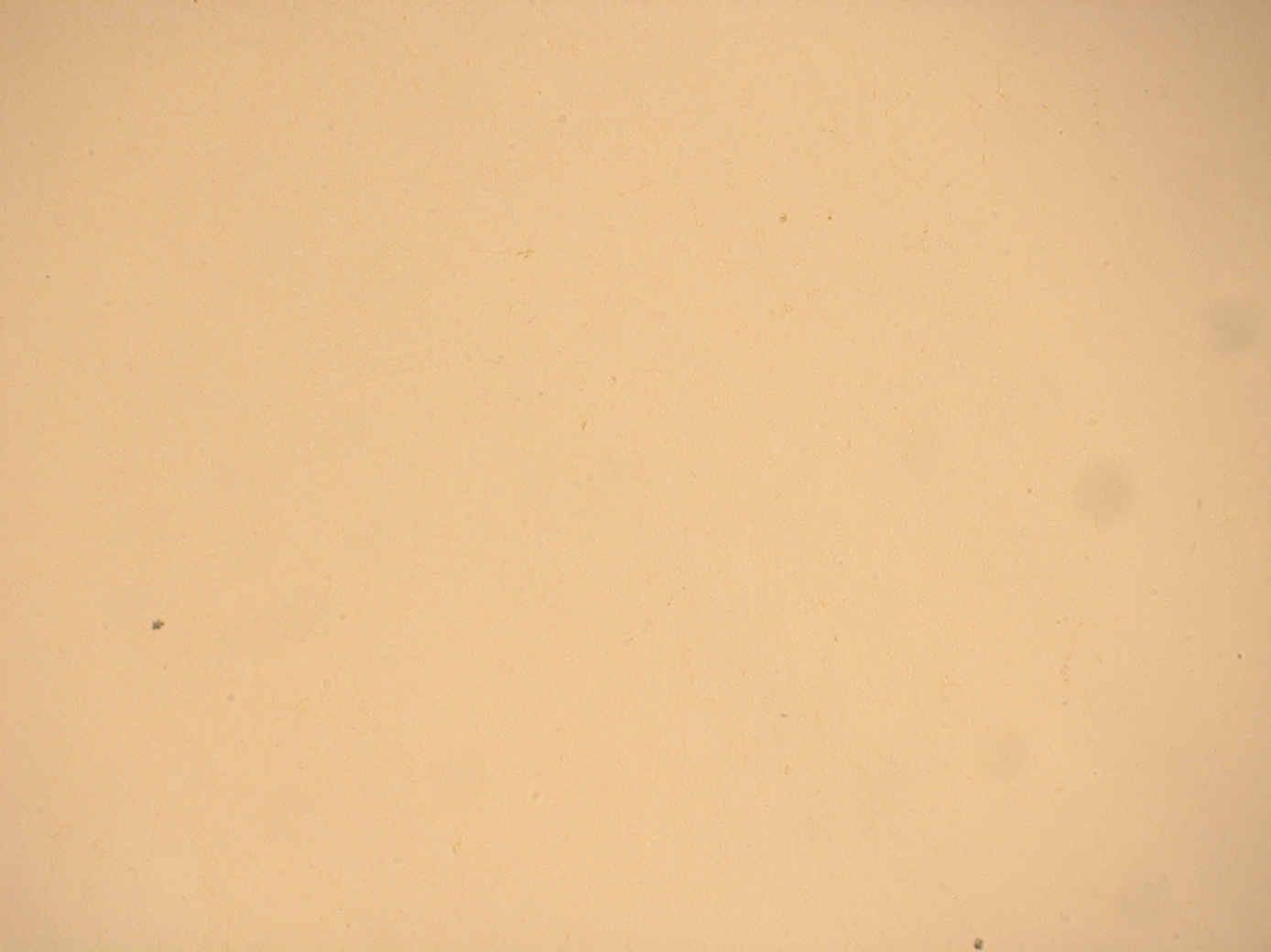

Supplement: Supplementary file 6 [file Image1.TIF]

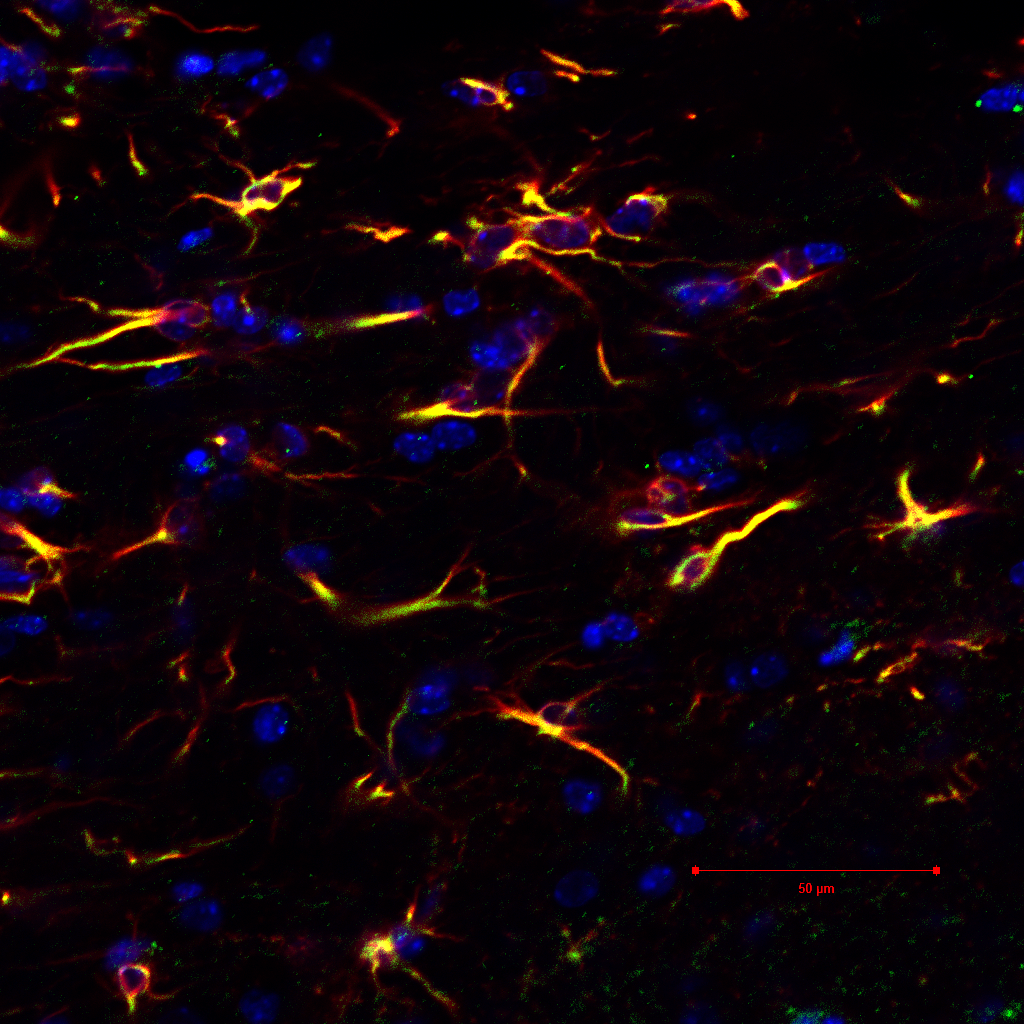

Supplement: Supplementary file 7 [file Image7.TIF]

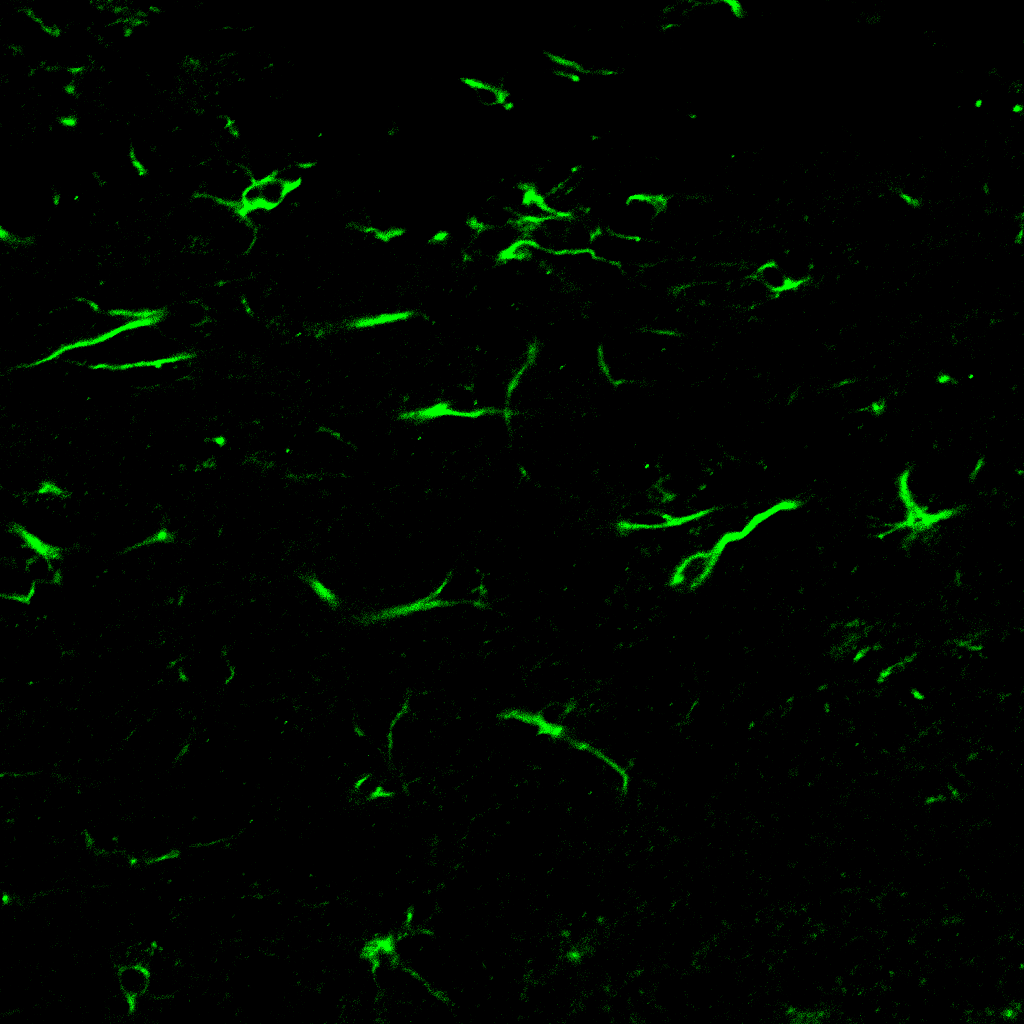

Supplement: Supplementary file 9 [file Image5.TIF]
