# Supplementary material for: Suppressing NK Cells by Astragaloside IV Protects Against Acute Ischemic Stroke in Mice Via Inhibiting STAT3
Source: Front Pharmacol. 2022 Feb 3;12:802047. doi: 10.3389/fphar.2021.802047 (PMC8852846; doi:10.3389/fphar.2021.802047)
Supplement: Supplementary file 8 [file DataSheet2.docx]

**Figure 2B:**

FL4: CD3, FL1: NK1.1

**
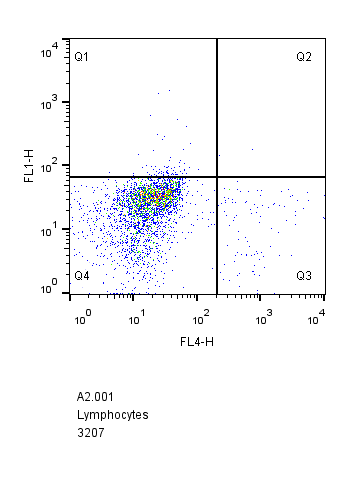
**

**MCAO+ASIV**

**MCAO**

**Sham**

**
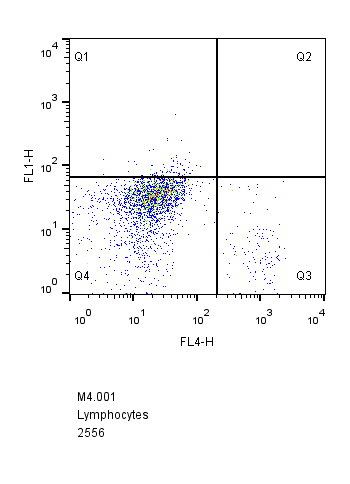

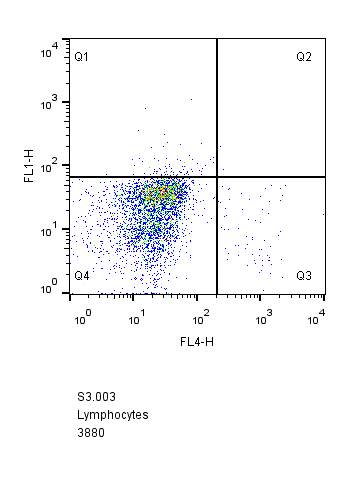
**

**Figure 3A:**

FL4: CD3, FL1: NK1.1

**PK136**

**
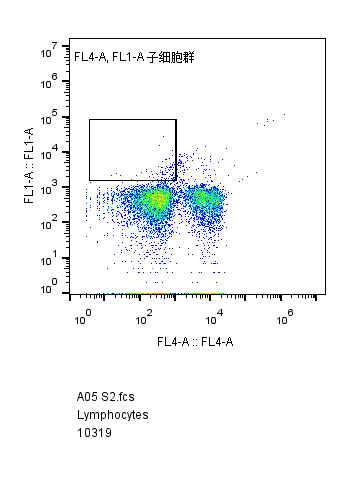

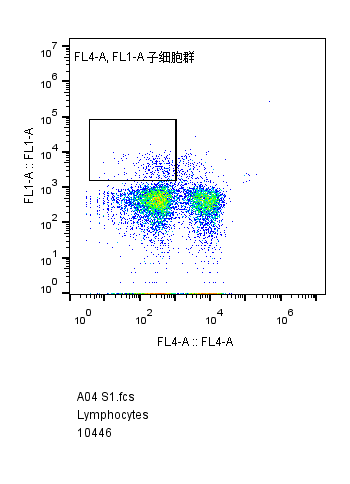
**

**Figure 5C:**

This experiment was performed and analyzed using CytoFLEX (BECKMAN COULTER). To be consistent with other results performing on BD FACS Calibur flow cytometer, we used Flowjo to transfer the data to present in the paper.

CytoFLEX PE: IFN-γ

**MCAO**

**MCAO+ASIV**

**Sham**


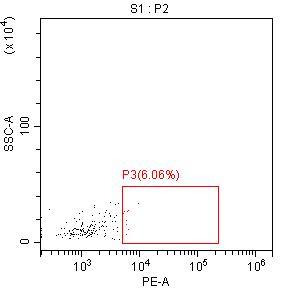

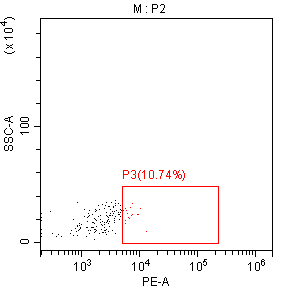

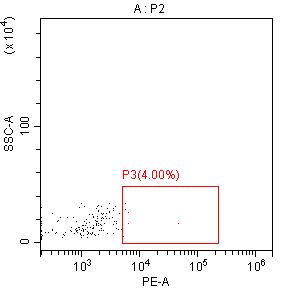


Transferred by Flowjo FL2: IFN-γ

**MCAO+ASIV**

**MCAO**

**Sham**

**
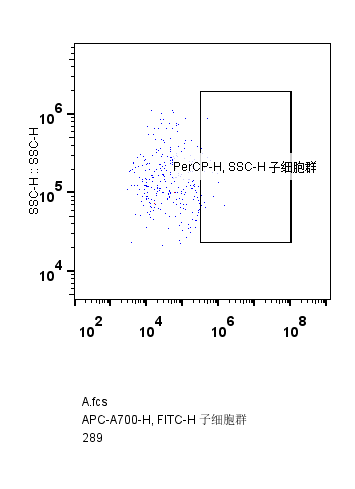

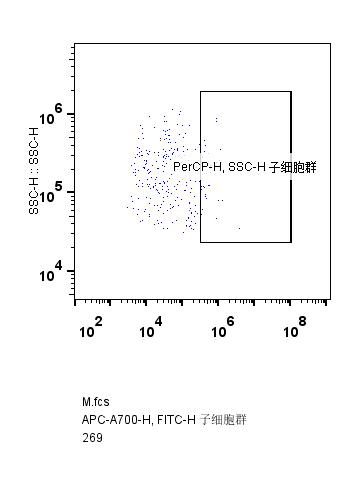

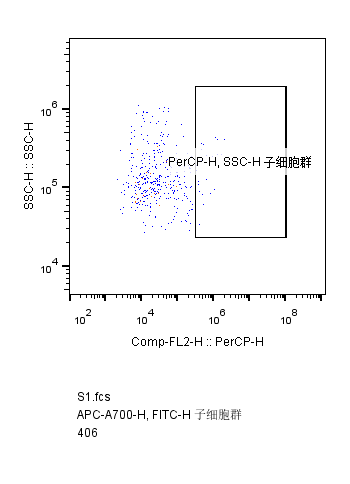
**

**Figure 6A:**

FL4: CD3, FL1: NK1.1

**
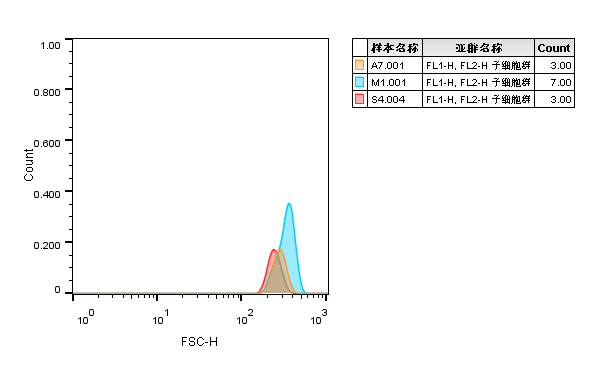
**

**
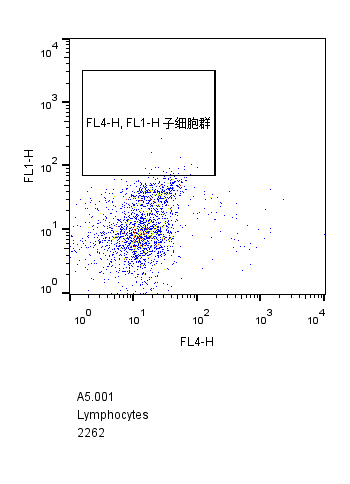
**

**Figure 6C:**

This experiment was performed and analyzed using CytoFLEX (BECKMAN COULTER). To be consistent with other results performing on BD FACS Calibur flow cytometer, we used Flowjo to transfer the data to present in the paper.

CytoFLEX FITC: NK1.1, APC: NKG2D

**OGD+ASIV**

**OGD**


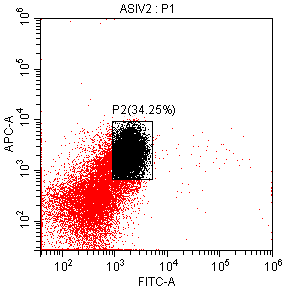

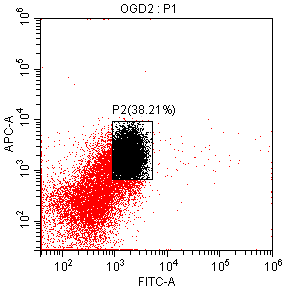


Transferred by Flowjo FL1: NK1.1, FL4: NKG2D

**OGD+ASIV**

**OGD**

**
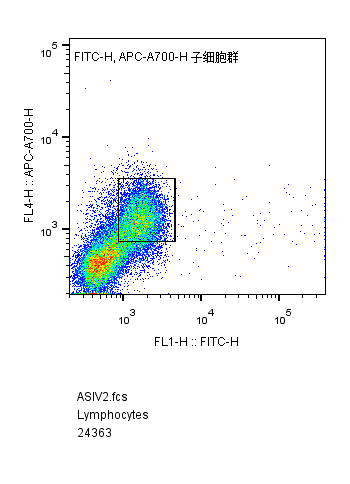

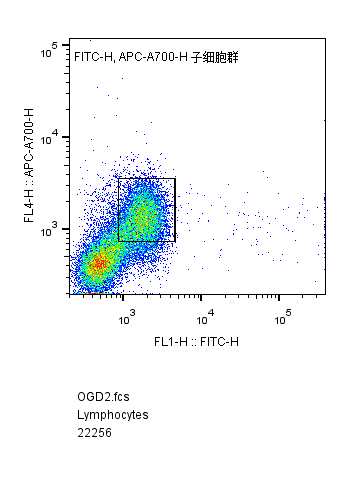
**

**Figure 6E:**

FL1: NK1.1, FL4: NKG2D

**OGD+ASIV**

**OGD**

**
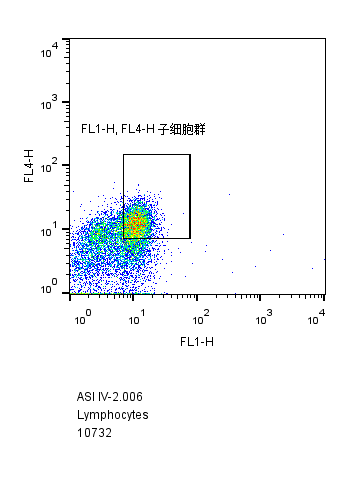

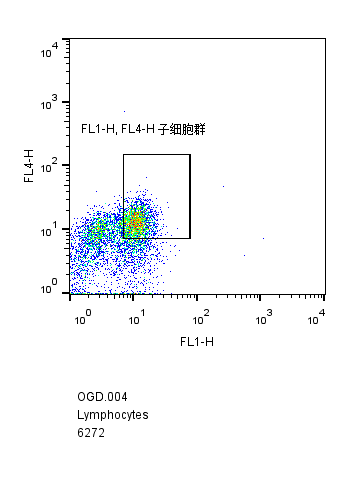
**

**Figure 7D:**

FL4: CD3, FL1: NK1.1

**MCAO**

**MCAO+ASIV**

**Sham**

**
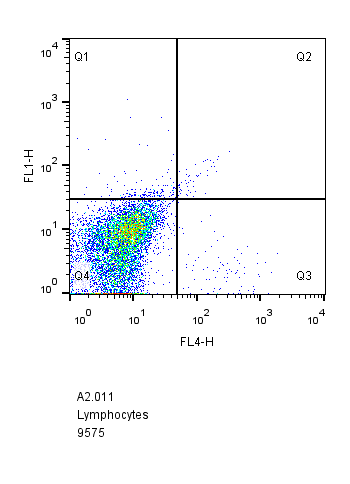

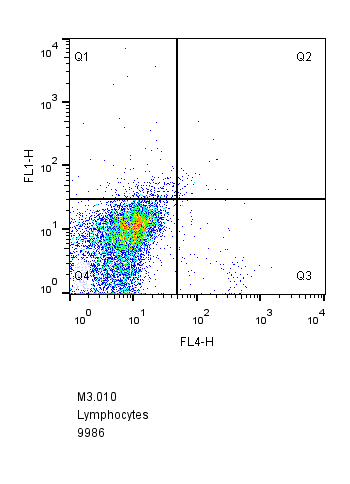

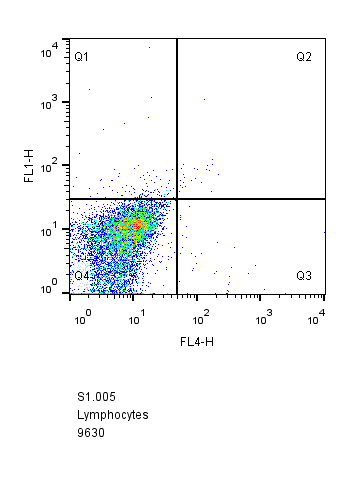
**

**MCAO+ASIV+CT**

**MCAO+CT**

**
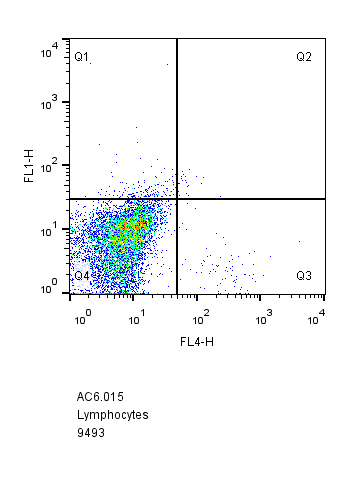

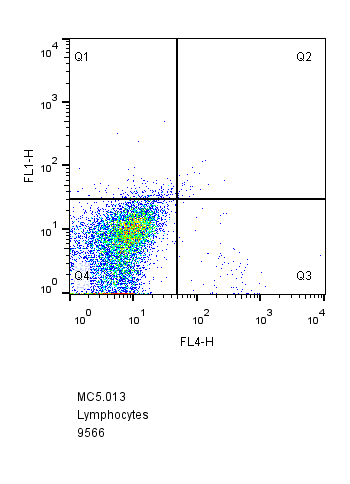
**

**Figure 8E:**

FL4: CD3, FL1: NK1.1

**
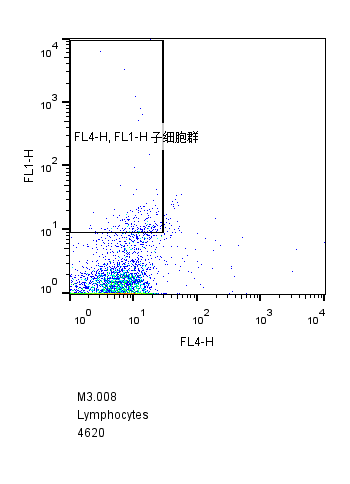

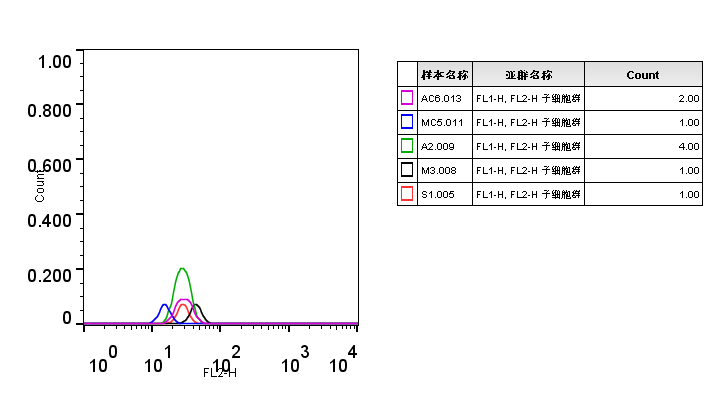
**
